# Network analysis of temporal functionalities of the gut induced by perturbations in new-born piglets

**DOI:** 10.1186/s12864-015-1733-8

**Published:** 2015-07-29

**Authors:** Nirupama Benis, Dirkjan Schokker, Maria Suarez-Diez, Vitor AP Martins dos Santos, Hauke Smidt, Mari A Smits

**Affiliations:** Host Microbe Interactomics, Wageningen University, Wageningen, The Netherlands; Wageningen UR Livestock Research, Wageningen University, Wageningen, The Netherlands; Systems and Synthetic biology, Wageningen University, Wageningen, The Netherlands; Lifeglimmer GmbH, Berlin, Germany; Laboratory of Microbiology, Wageningen University, Wageningen, The Netherlands; Central Veterinary Institute, Wageningen University, Wageningen, The Netherlands

**Keywords:** Gene expression, Microbiota, Data-integration, Long-term effects, Early life perturbations, Antibiotic, Stress, Pig intestine

## Abstract

**Background:**

Evidence is accumulating that perturbation of early life microbial colonization of the gut induces long-lasting adverse health effects in individuals. Understanding the mechanisms behind these effects will facilitate modulation of intestinal health. The objective of this study was to identify biological processes involved in these long lasting effects and the (molecular) factors that regulate them. We used an antibiotic and the same antibiotic in combination with stress on piglets as an early life perturbation. Then we used host gene expression data from the gut (jejunum) tissue and community-scale analysis of gut microbiota from the same location of the gut, at three different time-points to gauge the reaction to the perturbation. We analysed the data by a new combination of existing tools. First, we analysed the data in two dimensions, treatment and time, with quadratic regression analysis. Then we applied network-based data integration approaches to find correlations between host gene expression and the resident microbial species.

**Results:**

The use of a new combination of data analysis tools allowed us to identify significant long-lasting differences in jejunal gene expression patterns resulting from the early life perturbations. In addition, we were able to identify potential key gene regulators (hubs) for these long-lasting effects. Furthermore, data integration also showed that there are a handful of bacterial groups that were associated with temporal changes in gene expression.

**Conclusion:**

The applied systems-biology approach allowed us to take the first steps in unravelling biological processes involved in long lasting effects in the gut due to early life perturbations. The observed data are consistent with the hypothesis that these long lasting effects are due to differences in the programming of the gut immune system as induced by the temporary early life changes in the composition and/or diversity of microbiota in the gut.

**Electronic supplementary material:**

The online version of this article (doi:10.1186/s12864-015-1733-8) contains supplementary material, which is available to authorized users.

## Background

Evidence is accumulating that perturbations of the early life colonization of the gastro-intestinal (GI) tract by microbes induce long-lasting health effects in individuals [1, 2]. Though these effects have been studied and documented, the system components involved in the induction and maintenance of such long-lasting effects have not yet been studied in detail. This is because the GI tract itself is a complex and dynamic system with variable interactions between the host tissue, resident microbiota and nutritional factors. The host tissue is comprised of different cells with different functions, varying from the digestion and uptake of nutrients to providing resistance towards toxic components in the diet. The mucosal layer of the GI tract separates the lumen of the digestive tract from the rest of the body and contains the largest repertoire of immune cells that display a pleiotropy of immune signalling and response functions. In addition, the mucosal layer also generates endocrine responses to the lumen of the gut and to the rest of the body. The lumen of the GI tract harbours a complex ecosystem of a huge number and a large variety of micro-organisms, collectively called “microbiota” [3].

**Table 1:** Description of the 17 hubs in the three functional interaction networks

	Gene symbol	Function	Summary
OnlyTr1 hubs	GRB2 (17)	Important link between growth factor receptors on the cell surface and Ras signalling	Cell cycle/ Proliferation
	STAT3 (15)	In response to cytokines and growth factors, STAT family members are phosphorylated and translocate to the nucleus to function as transcription factors	Immune
	CDC42 (12)	A GTP-ase involved in signalling for several processes, cell migration, morphology, endocytosis, cell cycle progression and cell proliferation	Cell cycle/ Proliferation
	CAV1 (11)	Encodes a scaffolding protein that is an essential part of caveolar membranes	Immune
	FOS (11)	The FOS family encodes for leucine zipper proteins. Regulates cell proliferation, differentiation, transformation and apoptotic cell death	Cell cycle/ proliferation
Tr1&Tr2 hubs	MYC (31)	Transcription factor that activates growth related genes	Cell cycle/ Proliferation
	MAPK14 (24)	Important for the cascades of cellular responses evoked by extracellular stimuli leading to direct activation of transcription factors	
	RELA (24)	Forms a complex with NFKB transcription factor and regulates the NFKB pathway	Immune
	UBE2D2 (24)	Ubiquitin conjugating enzyme that catalyses covalent attachment of activated ubiquitin to other ubiquitin ligases	Ubiquitination
	ITCH (23)	Ubiquitin-protein ligase which accepts ubiquitin from an ubiquitin-conjugating enzyme and then directly transfers the ubiquitin to targeted substrates	Ubiquitination
	IKBKG (22)	Regulatory subunit of the IKK core complex which phosphorylates inhibitors of NFKB and ultimately the degradation of the inhibitor	Immune
	SP1 (22)	Transcription factor (activator or repressor) that regulates many cellular processes	Cell cycle/ Proliferation
	RHOA (20)	Regulates signalling from plasma membrane receptors to the assembly of focal adhesions and actin stress fibers	Cell cycle/ Proliferation
	RPS3 (20)	Component of the 40S ribosomal subunit, where translation is initiated	
	FBXW7 (19)	Part of the ubiquitin ligase complex (SCF) which recognizes and binds to phosphorylated targets	Ubiquitination
OnlyTr2 hubs	UBA52 (20)	One of the four genes that code for ubiquitin, and ribosomal components which are part of the ribosome 60S subunit	Ubiquitination
	STAT1 (12)	In response to cytokines and growth factors, STAT family members are phosphorylated and translocate to the nucleus to function as transcription factors	Immune

The number of connections of each gene in their respective network is given in brackets along with the gene symbol. The information about the genes is adapted from www.genecards.org. The hubs can be clustered into three broad groups, given in the last column, based on their functions. All these genes have significantly different time profiles in the treatment compared to the control

**Table 2:** Summary of the three correlation networks with information on bacterial groups

Bacterial groups	Number of correlated genes in			Information
	Only Tr1	Tr1& Tr2	Only Tr2	
*Eubacterium* et rel	14	165	128	Anaerobic, Gram positive, broad range of species
*Ruminococcus bromii* et rel	39	65	63	Anaerobic, Gram positive, keystone species in the gut, digestion of resistant starch
*Faecalibacterium* et rel	35	40	43	Obligate anaerobe, Gram negative, produces butyrate, has anti-inflammatory properties
*Campylobacter*	1	35	62	Microaerophilic, Gram negative, known pathogen in humans
*Lawsonia intracellularis* et rel	15	22	9	Gram positive, Porcine intracellular enteropathogen
*Butyrivibrio crossotus* et rel	22	1	2	Anaerobic, Gram positive, butyrate producer
*Bacteroides distasonis* et rel	5	1	5	Obligate anaerobe, Gram negative, reclassified to *Parabacteroides distasonis*, produces acetate and succinate, possible pathogen
*Fusobacterium* et rel		1	9	Anaerobic, Gram negative, generally butyrate producers, possible pathogen, potent lipopolysaccharide
*Eubacterium hallii* et rel	3			Anaerobic, uses lactate and produces butyrate
*Roseburia intestinalis* et rel	1			Anaerobic, Gram positive, butyrate producer
*Coprococcus eutactus* et rel	16		1	Anaerobic, Gram positive, mostly acetate producer
*Brachyspira*			43	Anaerobic, Gram negative, some known pathogens
*Eubacterium biforme* et rel			36	Anaerobic, involved in lipid metabolism
*Catenibacterium* et rel			32	Obligate anaerobes, Gram positive, digest simple sugars
*Turneriella*			18	Obligate anaerobes, Gram negative
*Bordetella* et rel			15	Obligate anaerobes, Gram negative, known to cause respiratory diseases
*Erysipelothrix*			11	Facultative anaerobes, porcine pathogens for skin diseases
*Lactobacillus acidophilus* et rel			10	Microaerobic, Gram positive, ferments sugars into lactic acid, probiotic
*Desulfovibrio* et rel			8	Aerotolerant, Gram negative, reduce toxic substances like sulphate
Uncultured *Prevotella*			4	Gram negative, pathogens in respiratory tract, breaks down proteins and carbohydrates
*Oxalobacter* et rel			2	Anaerobic, oxalate reducing
*Thiocapsa* et rel			2	Mostly anaerobic, oxidise sulphur, photosynthetic

The bacterial groups that are part of the networks are listed along with the number of genes with which they are highly correlated. Several genes are shared between bacterial groups. Both the genes and the bacterial groups are significantly different either in time or treatment between the control. The first seven groups are common between the three networks. Some general information on these bacterial groups is also given in the last column

Gut microbiota play an important role in modulating diverse gastrointestinal functions, ranging from enzymatic digestion to modulation of immune responses [4–10]. In turn, the host immune system has a regulatory effect on the composition and diversity of the microbiota [11], by mechanisms known as immune exclusion [12]. Early life colonization of the gut with microbiota is an important driver for the development and ultimate functionality of the GI tract in mammalians [8, 13, 14]. Long-lasting effects on the host due to disruption of early life colonisation of the gut have been demonstrated on the level of disease susceptibility, immune parameters [15] and the composition and diversity of microbiota [16]. Early life environmental factors, such as caesarean delivery [17], breastfeeding [18–20], exposure to stress [21, 22], and the use of antibiotics [23, 24] influence the microbial colonisation of the gut and affect the development and programming of the mucosal and systemic immune system [16, 25]. Such early life factors may also result in variation of the abilities of the microbiota to ferment carbohydrates into short-chain fatty acids [26] and/or to ferment indigestible proteins in later stages of life.

The effect of the use of antibiotics on the physiology of the host is believed to be due to the primary effect of antibiotics on the loss/change in bacterial (sub)-populations, especially in the GI tract [27]. The spectrum of the antibiotic and its mode of action determines the effects it has on the gut microbial community [27]. Some reports in this area also suggests that antibiotics have a direct effect on the immune system of the host [27, 28]. For young piglets it has recently been demonstrated that an antibiotic treatment at day 4 after birth causes changes in microbial populations and in tissue gene expression patterns in the small intestine [29, 30]. Stress is another factor that can influence the functionality of the GI tract by a temporary secretion of hormones. Such short term hormonal secretions may cause several long lasting effects on the GI immune system [31–33] and on microbiota composition [34]. This has led to studies focusing on the brain-gut-enteric microbiota axis [35–37]. The effect of stress on the GI tissue is most obvious in changes of morphology and functions of the gut [38–40].

The first objective of this study was to develop a workflow that can be used to analyse biological data in two dimensions simultaneously, over time and between treatments. The second objective was to apply this workflow on two types of gut-related data as measured in an experiment with pigs exposed to an early life perturbation, followed by effect measurements at three different time-points later in life. Using these methods we endeavour to identify gut system components that contribute to induction and maintenance of long lasting effects of early life perturbations. We aim to find major host- and microbe-related components that propagate or regulate these long term effects. Such components may form potential targets to modulate early life events that affect later life immunologic performance. We used piglets as a model and the exposure to an antibiotic and/or stress on day four after birth as the perturbations. We measured whole genome gene expression profiles of intestinal tissues and provided community scale data on the composition and diversity of microbiota on three different time-points, taken during different stages of the life-span of pigs, neonate, adolescent and full grown adult. In order to take into account the extreme changes in the physiology and morphology of the animal, we simultaneously studied the effect of the treatment and the time. Once the gene expression and microbiota data were analysed separately, we integrated both to obtain information on their possible interactions.

## Methods

### Experimental design

The animal experiment consisted of one control group and two treatment groups, each consisting of 48 piglets derived from 16 different sows (TOPIGS20 (GY × NL)). Each litter contained 4 piglets of each treatment and control group. Litter-mates stayed with their sow until weaning at day 25. After weaning, the same number of piglets of each treatment and control group were housed together in pens. All pens were located in the same compartment. The first treatment group (Tr1) was given a dose of Tulathromycin (0.1 ml, 2.5 mg/kg body weight) on the 4^th^ day after birth and then left undisturbed until the time of tissue sampling after sacrifice. The same dose of the antibiotic was given to the second treatment group (Tr2) on the 4^th^ day after birth but these piglets were also subjected to stressful conditions on the same day. The stress was in the form of handling of the piglets, which is common practice in intensive pig husbandry systems (eg, weighing, nail clipping). The control group (Ctrl) was not disturbed for the entirety of the experiment until the time of sacrifice for sampling.

Sampling was done on three time-points, day 8 after they were born, day 55 and day 176. On each of these days, 16 piglets from each treatment group and derived from 16 different sows were sacrificed and samples were collected for microarray and microbiota analysis. Further details of the experiment have been described elsewhere [29]. The experiment was approved by the institutional animal experiment committee “Dier Experimenten Commissie (DEC) Lelystad” (2011077.b), in accordance with the Dutch regulations on animal experiments. Additional file 1: Figure S1 gives an overview of the experimental design.

### Sample preparation and data generation

For transcriptome analysis, jejunal tissues scrapings were taken and RNA was extracted from the samples for microarray analysis as described in [29]. For microbiota analysis luminal contents were taken from the same location of the jejunum as the tissue scrapings and microbial DNA was extracted. The microbial composition was detected by the Pig Intestinal Tract Chip (PITChip) [41] version 2.0. The PITChip is a phylogenetic microarray with 3299 oligonucleotides based on 16S rRNA gene sequences of 781 porcine intestinal microbial phylotypes [42, 43]. The protocol for hybridization and generation of data was performed essentially as described before [29, 44]. More details on sample preparation is given in previous descriptions of this experiment [29] and the data is available in GEO [45] with the accession number GSE53170 [46].

### Gene expression analysis

#### Microarray normalization and quality control

Background correction and quantile normalisation was performed on the microarray data (GSE53170) using the R package LIMMA [47] from Bioconductor [48]. After quality control, six microarray samples were removed from the original 72 samples and all further analysis were done on the remaining 66 microarray samples. Data points below the 5^th^ percentile of intensities were removed, this resulted in 25,915 genes from 66 microarray samples. Details of the applied analysis pipeline are given in Additional file 2: Figure S2.

#### Identification of differences in gene expression profiles

To identify genes with significant expression profile differences over time between the experimental groups, we used maSigPro [49] from Bioconductor. The time profile (expression over the three time-points) from each treatment was compared to that of the control. Quadratic regression was used to retrieve genes with time profiles significantly different for the treatments versus the control group. The calculations were done with the default settings of the function maSigPro. With three time-points there are three regression coefficients calculated for each gene’s expression profile. At least one of these coefficients has to be significant (with FDR corrected p-values less than 0.05) for the gene to be included in the result. The first coefficient (β1) denotes the difference in the first time point (day 8) between one treatment group and the control. The second one (β2) indicates the difference in slope between the first two time points. The third coefficient (β3) shows difference in curvature of the expression patterns and can thus capture long term differences between treatment and control expression patterns of the same gene. Each treatment is compared against the control group and this gives two lists, Tr1vsCtrl (Antibiotic vs Control) and Tr2vsCtrl (Antibiotic + Stress vs Control). For the rest of the analysis three lists of genes were used; genes unique to Tr1vsCtrl (**OnlyTr1**); gene unique to Tr2vsCtrl (**OnlyTr2**) and the overlapping genes (**Tr1&Tr2**).

GO (Gene Ontology) enrichment analysis [50], with focus on the sub-ontology Biological Process, was performed using the R package topGO [51]. The Fisher test was applied to obtain significantly enriched GO terms, only the terms with p-values below 0.01 were included.

#### Functional interaction networks

Functional interaction networks among groups of genes selected from the data, were built using the Cytoscape [52] Reactome FI (Functional Interaction) application [53–55] and visualised with the organic layout. The network was built from information in the Reactome FI database such that nodes are genes and edges are interactions, either known or predicted. Modules in the networks were identified using community structure detection as described in [56]. Using inbuilt functions in the Reactome FI app, GO enrichment analysis was performed on each of the identified modules. The final networks consisted of modules with more than 5 nodes and with significantly enriched GO terms. The topological features of these final networks were determined using the NetworkAnalyzer [57] tool in Cytoscape. Hubs were defined to be the nodes in the top 40 % of the degree distribution, where the degree is the number of connections of each node. These hubs were analysed using information from www.genecards.org.

### Analysis of microbiota

Microbial populations from the jejunum were analysed using the PITChip.2 [41], which provides information on three levels of taxonomic resolution, level 1 is similar to the phylogenetic family, level 2 corresponds mostly to the genus level and level 3 is more the species level. Level 2 data provided population percentages for 151 microbial genus groups. Initial exploratory analysis was done on the microbial data with the R package microbiome (http://microbiome.github.io/) and the MySQL database as described by Rajilic-Stojanovic [44]. Microbial groups that do not differ between control and treatment piglets were filtered out with a threshold of 0.01. A two-way ANOVA analysis of the temporal patterns with the package maSigPro was used to obtain only the groups that change with treatment or time, with a statistical significance threshold of 0.05.

### Integration of gene expression and microbiota data

The mixOmics R package [58, 59] was used to integrate gene expression and microbiota abundance data and to perform regression analysis of these two data types. The genes used were those selected using the method described in section Gene expression analysis, which leads to the identification of genes whose temporal expression profiles were significantly different between the control vs. the treatment group. The bacterial groups used for integration were also different either across time or between treatments. We set the microbial information as the independent variable and the gene expression data as the dependent variable. Interchanging the two set of parameters did not affect the results significantly. We only used the significant genes from the gene expression analysis as input for the data integration. Subsequently three networks were built using sparse Partial Least Squares for each of the gene lists **OnlyTr1**, **OnlyTr2** and **Tr1&Tr2**. The first two networks were based on six variables (treatment and control in three time-points) and the third one on nine variables (two treatments and the control in three time-points).

The resulting un-directed network connects the microbial groups and the genes that have absolute correlation values above 0.8. A positive high edge weight hints to a positive regulatory relationship while a low (negative) weight indicates a possible repression relationship. Networks were visualised using Cytoscape 3.1.0. Enrichment analysis was done on the gene neighbours of the bacterial groups via topGO. Fisher’s test was applied on the results and only terms with p-values lesser than 0.01 were further analysed. The functionality of the bacterial groups was determined with the help of experts in the field.

## Results

### Genes, gene networks and hubs

**Fig. 1:**
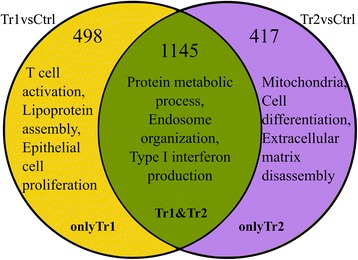
Summary of GO Enrichment analysis results from topGO. Biological processes (Gene Ontology terms) are given based on manual interpretation of the most significantly enriched terms obtained with topGO. The two circles represent the Tr1vsCtrl and Tr2vsCtrl comparisons. Numbers denote the number of input genes in topGO, these genes are have significantly different time profiles in the treatment vs the control groups. In the yellow, green and purple fields enriched processes are given for OnlyTr1, Tr1&Tr2, and OnlyTr2, respectively

For gene expression analysis, the temporal profiles of the samples from each treatment group were compared with those of the control group. We used quadratic regression analysis to obtain two gene lists: one for Tr1vsCtrl (1643 genes) and another one for Tr2vsCtrl (1562 genes). These lists are in Additional file 3. The genes in these lists show significant differences in their gene expression profiles over time between the control and treatment groups. Additional file 4: Figure S4 shows the temporal expression profiles of some genes, showcasing different possible behaviours under different conditions. For example, the expression profiles over time of CHIT1 differs for all three conditions while the profiles of some other genes, such as GRB2 and MAPK14, show most difference in only one treatment compared to the Ctrl. In other cases, such as ERBB4, MX2 and RELA, significant differences in expression are restricted to a single time point. Based on the β3 coefficients (explained in the Methods section Gene expression analysis), 60 % of the **OnlyTr1** and **OnlyTr2** gene-lists have genes with long lasting differences between the treatment and control. The **Tr1&Tr2** list has 91 % of the genes that have significant long term differences. The three gene lists; **OnlyTr1**, **Tr1&Tr2** and **OnlyTr2** consist of genes that have gene expression profiles over time that are significantly different in the treatment compared to the control group. These lists are used for the follow-up of the analyses. On these three lists, functional analysis was done using GO enrichment analysis for biological processes with topGO. A summary of the results is presented in Fig. 1.

**Fig. 2:**
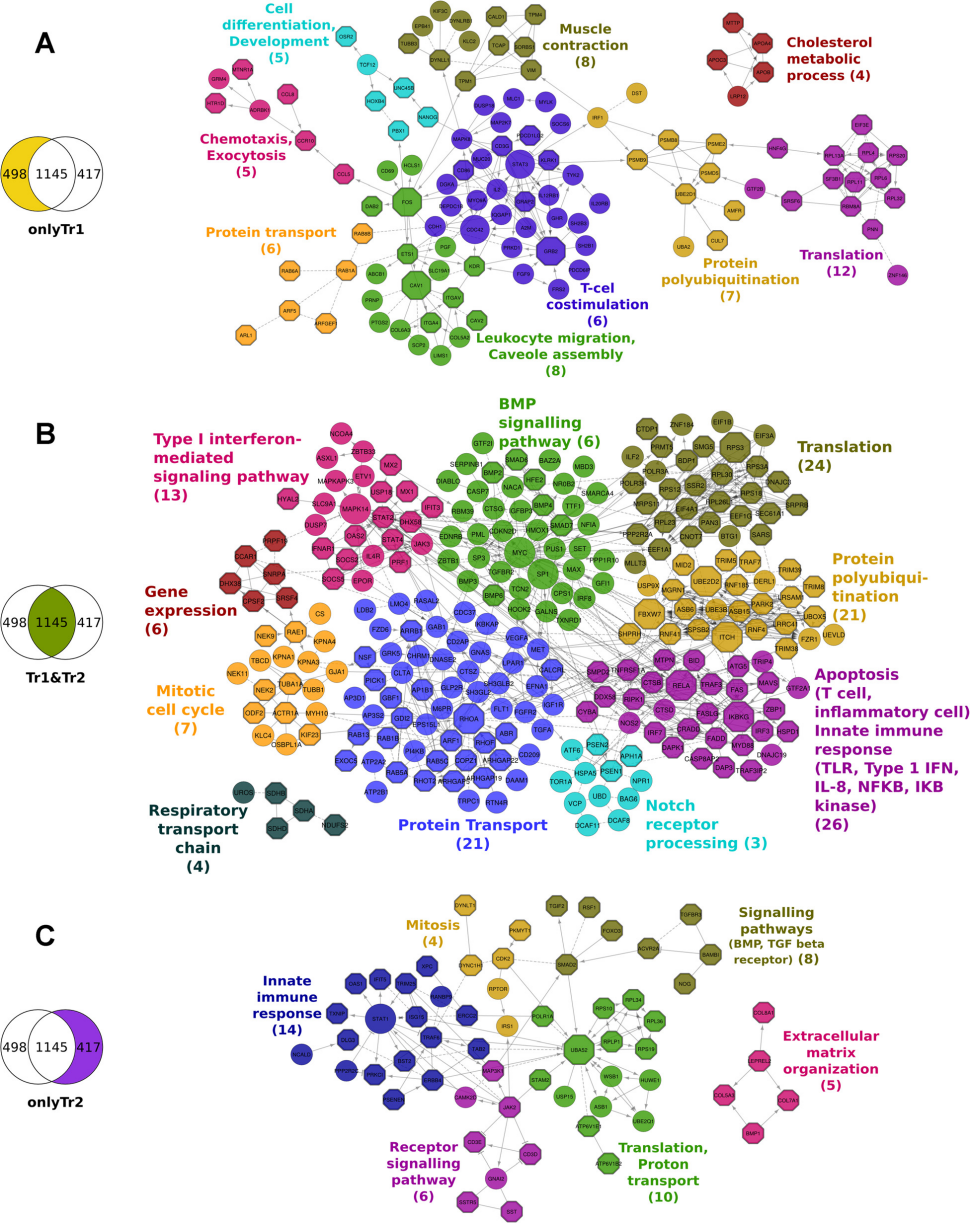
Functional Interaction networks (**a**, **b**, **c**). Genes are represented as nodes in the networks, all these genes have time profiles that are significantly different in the treatment than in the control. The edges represent interactions between genes as determined by Reactome. Arrows represent directed interactions, bar-headed arrows indicate inhibition reactions. Dotted lines indicate predicted relationships. Network A was built from **OnlyTr1** genes, network B from the common or overlapping genes (**Tr1&Tr2**) and network C from **OnlyTr2** genes. Colours in the network represent the network segmentation into modules. The text denotes the GO term that was most enriched for the genes in that module and the number in brackets denotes the number of genes associated with that particular GO term. Octagonal nodes are related to the GO term at the set p-value threshold. The nodes with a larger diameter are hubs in the networks. High resolution images of the individual networks are given as Additional file 5: Figure S5, Additional file 6: Figure S6 and Additional file 7: Figure S7. The nodes of the **Tr1&Tr2** network were rearranged for better visualisation of the modules; the network in the original structure is in Additional file 8: Fig. 4 Additional file 9: Fig. 5

In order to verify the results of the topGO analysis and to get insight into networks of genes potentially involved in the induction and maintenance of the long-lasting effects, a network based analysis was used. The Reactome Functional Interaction (FI) database was used to build three functional interaction networks, one for each list of genes. In any of the explored gene sets, about 30 % of the genes were represented in the FI networks. More than 50 % of the nodes had significant long-term differences from the control, with the Tr1&Tr2 FI network having the biggest fraction (94 %). Within each of the FI networks, we were able to identify several topological modules. We identified 9 modules with significant biological function in the **OnlyTr1** network, 10 modules in the **Tr1&Tr2** network, and 6 modules in the **OnlyTr2** network. The modules are depicted by different colours in Fig. 2. Subsequently GO enrichment analysis for Biological Processes was performed on the genes in each of the identified modules. The GO terms with the highest enrichment score for each module are shown in Fig. 2. The results of GO enrichment by both methods for the same gene lists are notably different especially in the **OnlyTr2** list. Information on the various topological parameters of the three networks can be found in the Additional file 10.

The results from topGO show that the **OnlyTr1** and **Tr1&Tr2** genes are mainly involved in innate and adaptive immune processes, whereas the **OnlyTr2** list is dominated by genes involved in developmental processes. Compared to the topGO analysis, the enrichment analysis from Reactome FI provided more detailed functional information. It showed that the genes in all three lists are involved in immune processes with dominance of adaptive immune processes in **OnlyTr1** and innate immune processes in **OnlyTr2.** The overlapping gene list **Tr1&Tr2** included immune signalling functions, interferon and interleukin genes, that are speculated to contribute to both types of immunity [60–62].

**Fig. 3:**
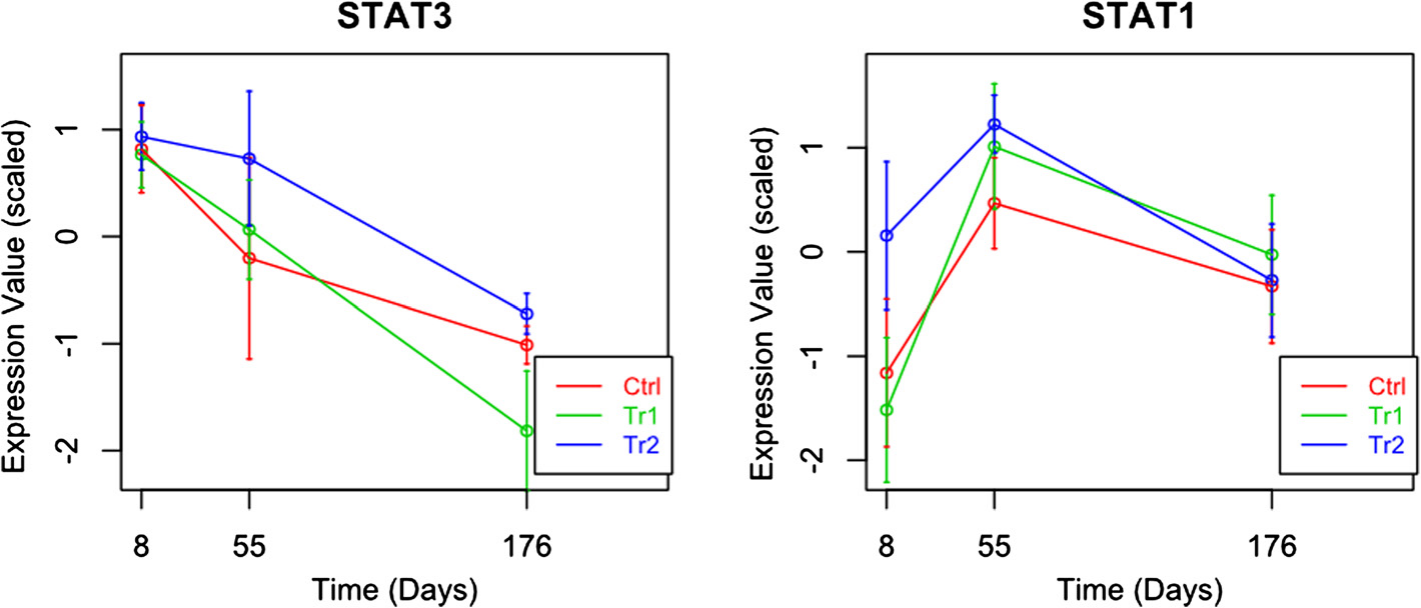
Gene expression patterns of important hubs. In Fig. 3 each graph depicts the temporal expression pattern of a single gene. These temporal changes are shown under three different conditions: Ctrl (red line), Tr1 (green line), and Tr2 (blue line). The x-axis indicates the time in days. The expression values (y-axis) are scaled such that the average expression of each gene is 0 and the standard deviation is 1

The network analysis provided insight into genes with potential high level regulatory activity, i.e. genes in the network with high number of connections/edges. A list of these potential high level regulators or hubs and a gist of their known biological functions is given in Table 1. The temporal expression patterns of three of these 17 genes are shown in Fig. 3 (all 17 of them can be found in the Additional file 11: Figure S11). Except for the two hubs of the **OnlyTr1** FI network, all the hubs have long term differences in expression between the treatment and control based on the β3 regression coefficient. The hubs in all the three networks can be roughly assigned to three functional categories: immune, cell cycle or proliferation, and genes involved in ubiquitination. There are two genes that are not part of these three clusters, MAPK14 and RPS3, where the latter is an important component of the ribosome. MAP kinases act as an integration point for multiple biochemical signals, involved in a wide variety of cellular processes like proliferation, differentiation and development. They are activated by environmental stresses or cytokines.

All the hubs in the **OnlyTr1** are either immune or cell cycle/proliferation related genes. This is reflected in the positioning of these genes in the network. They are found in the two modules related to immunity; T-cell co-stimulation and leukocyte migration (Fig. 2). The genes in the ubiquitin cluster are from the **Tr1&Tr2** and **OnlyTr2** networks; they code for ubiquitin itself (UBA52) and also for proteins that perform the conjugation and ligation of ubiquitin to other proteins. These hubs in the **Tr1&Tr2** network are in the module for protein ubiquitination; this module has the highest number of hubs. Other hubs in this network are from four modules as shown in Fig. 2. In the **OnlyTr2** network, there are two hubs, UBA52 and STAT1.

### Microbiota temporal changes

The relative contribution of the microbiota was filtered as described in Methods and was left with 125 microbial groups which represent 99 % of the population. The relative contribution values of these 125 groups were used in the regression analysis to identify groups that can be further related to gene expression levels. After a two-way ANOVA analysis in maSigPro, there were 46 microbial groups that showed significant differences over time or treatment compared to the control. Some bacterial groups like *Brachyspira* show different contributions in different conditions but some groups like *Bacillus* et rel show major differences only at one time point and one treatment. Additional file 12: Figure S12 shows these temporal changes for all the 46 groups.

### Integration of microbiota and gene expression analysis

**Fig. 4:**
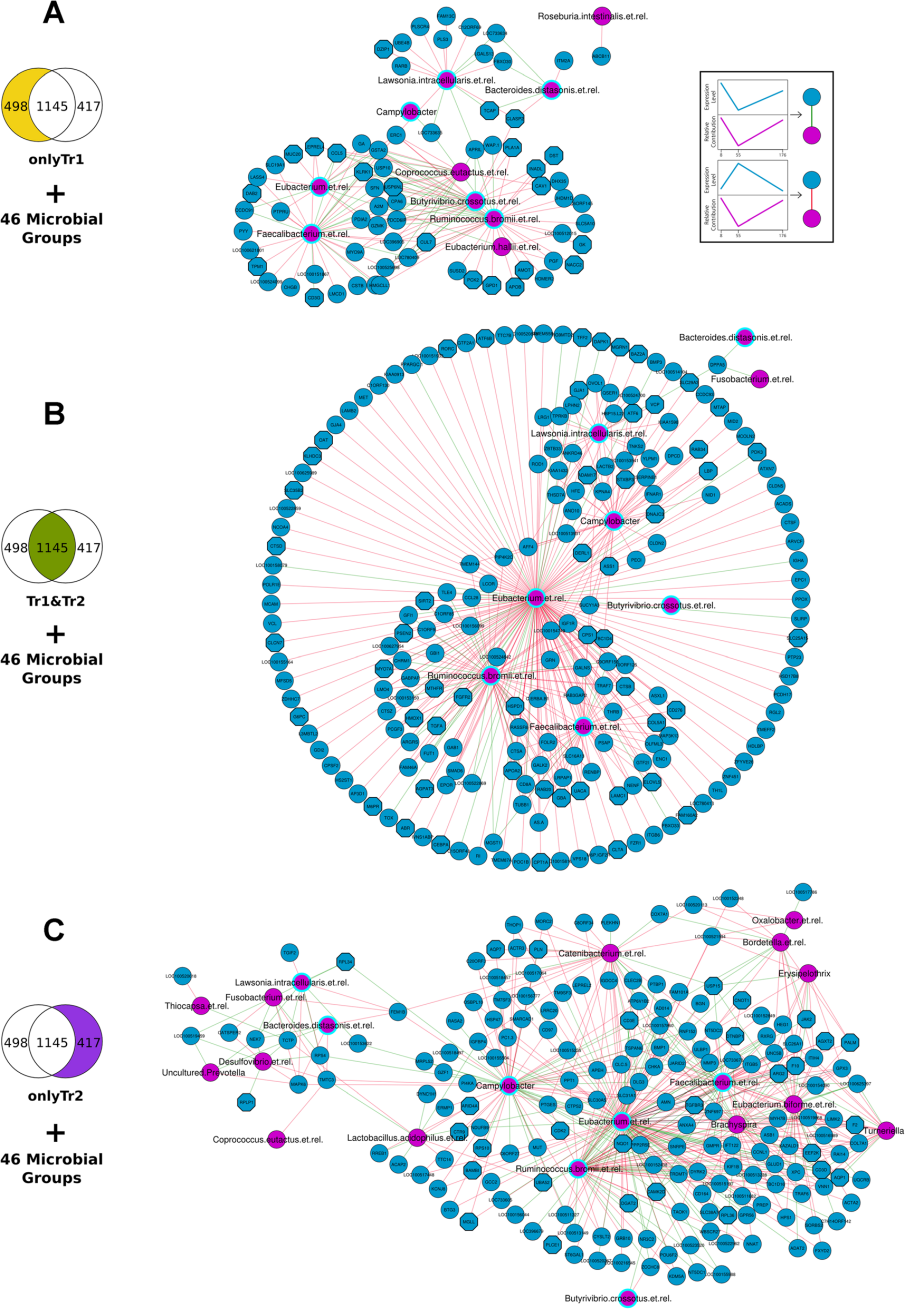
Correlation networks of changes in gene expression patterns and microbiota composition: Blue nodes represent genes and the pink ones represent bacterial groups; pink nodes with a cyan boundary are nodes common in the three networks. The edges represent positive (green) and negative (red) correlation between a gene and a bacterial group. Networks (**a**), (**b**) and (**c**) were built by correlating the gene lists **OnlyTr1**, **Tr1&Tr2** and **OnlyTr2** respectively with the 46 microbial groups resulting from the regression analysis. All the nodes (bacterial groups and genes) have a significantly different expression profile in time or treatment compared to the control. High resolution images of the individual networks are given as Additional file 10: Figure S10, Additional file 8: Figure S11 and Additional file 12: Figure S12

By performing statistical integration of both the microbiota and host gene expression datasets, (mixOmics R package), we tracked changes in jejunal gene expression which follow the changes in microbial populations as determined on the same location in the gut. The changes will reflect the similarity or dissimilarity of a pair of data points across time. The resulting similarity matrix was used to connect the microbial groups and the expression of genes into a network. The first network, **OnlyTr1**, was built with 498 genes in 6 conditions (Ctrl, Tr1 with 3 time-points each) and 46 level2 microbial groups in the same conditions. The second network (**Tr1&Tr2**) was built with 1145 genes and the same 46 microbial groups, and the conditions were the control and both Tr1 and Tr2 at the 3 time-points, this gave rise to 9 conditions. The third one had again 6 conditions (Ctrl and Tr2 in 3 time-points) with 417 genes and the same 46 bacterial groups. The networks represent the correlation between the microbiota and the gene expression data and are shown in Fig. 4.

There are a total of 22 bacterial groups involved in the three networks, among these, 7 are found in all three networks (Table 2). Most of these bacterial groups share the same temporal pattern of relative contribution (shown in Additional file 13) and are characterized by an increase in abundance over time. Of the 22 bacterial groups in the three correlation networks, only six are known to be possible pathobionts and their abundance, though increasing with time, remains low at all time-points and treatments. The other 16 bacterial groups are known for being beneficial to the intestine by producing short chain fatty acids or reducing toxic substances (Table 2). Four of the bacterial groups show a decrease in abundance, and are found in the **OnlyTr2** correlation network. Three bacterial groups had consistently large number of gene neighbours: *Eubacterium* et rel, *Faecalibacterium* et rel and *Ruminococcus bromii*. These three groups also share several gene neighbours in all the three networks and are quite central in the network as indicated by network parameters (Additional file 14).

Network topological features also revealed that in all three networks, 40 to 55 % of the host genes were neighbours of a single bacterial group. The rest of the genes highly correlate with more than one bacterial group, and a maximum of 8 bacterial groups. About 60 % of the genes are shared between at least two bacterial groups. The maximum radiality (indicative of how connected the nodes are to all the other nodes, with values ranging from 0 to 1) was 0.8 to 0.9. This is apparent from the visualization of the networks in Fig. 4 which shows only a few nodes in the centre connected to most of the other nodes. In the three correlation networks, 60–80 % of the edges have negative correlation values.

The GO terms that appeared enriched in the gene list of each of the correlation networks are mostly related to four broad functions, metabolic processes, transport of substances, translation, and some immune processes (shown in Additional file 15). Most of these genes are negatively correlated with the bacterial groups; this indicates general reduction of the expression levels of these genes across time. But these genes exhibit significant treatment effects, especially long term effects which are not reflected in the temporal patterns of the bacterial groups with which they are highly correlated.

In each correlation network, 20 % of the genes are found in the FI networks from gene expression data alone. A separate topGO analysis shows that the genes in each of the correlation networks are enriched for GO terms that are related to four broad categories of biological processes. These categories are, Metabolic Process, Transport, Translation and Immune Processes. In the **OnlyTr1** and **Tr1&Tr2** correlation networks, the genes associated with the most significant GO terms are mostly (70 and 80 %) negatively correlated with the microbial groups. But in the **OnlyTr2** correlation network, these genes are mostly (60 %) positively correlated with the microbial groups. More than 50 % of the genes in the three networks have significant long term differences from the control.

## Discussion

In this paper we describe a workflow and a set of methods capable of analyzing and integrating different types of data in two dimensions, treatment and time. These methods were then used for the identification of gut system components that contribute to the induction and maintenance of long-lasting effects in the GI tract as induced by perturbations at a young age. To this end we combined multiple, freely available tools and advanced statistical analytical tools. We used temporal gene expression patterns, obtained using microarray technology, to identify genes and biological processes that are affected over time by the early life perturbation with antibiotic and/or stress treatments. In addition, we used community-scale analysis of gut microbiota from the same location of the gut to identify changes in microbiota profiles over time due to the perturbations.

With this approach we have taken the first steps in unravelling the genomic and microbial networks that contribute to long-lasting responses of early life perturbations in the gut. The results reveal that there are significant long-lasting differences in the system of the GI tract between the different perturbations groups, mainly at the gene expression level. The data presented are consistent with the hypothesis that observed long lasting effects on gene expression are most probably due to differences in the programming of the gut immune system as induced by the temporary early life changes in the composition and/or diversity of microbiota in the gut. Furthermore, we were able to identify potential key regulator genes (hubs) for the long-lasting effects and we have identified microbial groups that are potentially associated with the observed changes in intestinal gene expression.

In the following sections of this Discussion we explain the rationale behind choosing these methods for our particular biological question. In the sections “Biological relevance of the hubs”, “Crosstalk between host and microbe components” and “Understanding the long lasting changes in the host” we examine the biological relevance of the results. The other sections mainly deal with methodology aspects.

### Experimental approach

Intestinal gene expression and microbiota profiles were generated on three different time-points; day 8 (neonatal), 55 (young adult), and 176 (adult). Day 8 was chosen because it is known that immediately after perturbations, as used here, changes occur in the pattern of microbial colonizing of the gut as well as in the GI tract mucosal gene expression [27, 28, 34]. The second measurement at day 55 was chosen because the microbiota would have stabilised by then after weaning (around day 28). Weaning is an extremely turbulent process [63–65] that brings about several temporary changes which are not the focus of our study. The last measurement was on day 176 after birth which coincides with the time of slaughter of these pigs.

Intestinal bacterial composition and gene expression patterns change over the lifetime of an animal due to changes in nutritional, environmental and physiological factors [64, 66, 67]. Obviously, these normal developmental changes could also be detected in this study; however we were particularly interested in deviations from the normal developmental patterns due to the early life perturbations. Previous work on subsets of these data [29, 30] showed that there are significant differences between the control and treatment groups at each time point. In the analysis described here, we incorporated the information across time and between treatments by the use of a quadratic regression analysis (maSigPro R package). In addition, we studied the behaviour of the microbiota over time and looked for correlations with gene expression patterns using the mixOmics R package, because it is known that the microbiota and gut mucosal tissue respond to each other in various ways [68, 69].

### Analysis of treatment effect on temporal gene expression

For analysis of data derived from three time-points and two treatments, a regression analysis is better suited than a pairwise comparison with linear models, PCOA or hierarchical clustering which was done in earlier work by Schokker et al. [29, 30]. The resulting gene lists from Tr1vsCtrl and Tr2vsCtrl are separated into three lists to be able to look at the biological processes common to both the gene lists which is expected to be due to the effect of the antibiotic. With the resulting three gene lists, we performed GO enrichment analysis with two different methods: topGO and Reactome. An important difference between the two methods is that Reactome is manually curated with experimentally verified information; whereas topGO only uses gene annotation information that is, in many cases, automatically generated without further manual verification. As expected, the Reactome analysis resulted in richer information and encompassed most of the results from the topGO analysis. The network format of Reactome also allows for more informative visualization and further analysis using the topology. For these reasons we decided to concentrate our gene expression analysis on the enrichment results from Reactome where ever possible.

### Network analysis

In the FI networks **OnlyTr1** and **OnlyTr2**, more than half of the nodes have a significant long-lasting expression pattern difference in the treatment versus control. This is significant for most of the nodes in the **Tr1&Tr2** FI network which is representative of the action of antibiotic. This reveals that most of the long lasting effects are due to the antibiotic effect in the treatment. These genes with long lasting differences are spread over all the modules which indicates that all the biological processes in the network (Fig. 2) are different over time. Several of these processes are essential cellular processes and some are immune related. This could lead to situations that the treated animals respond differently to external stimuli compared to control animals. In order to look further into these long lasting differences we analyse the hubs of the network.

### Analysis of hubs

Investigating the hubs of networks gives insight into the entire network function [70] and the nodes in our networks are also found to be important for several biological functions [70–72] as mentioned in the Results. In most network analysis the degree itself [73] or the degree distribution is used to choose the most connected hubs [74]. We chose the top 40 % of the degree distribution as hubs, which is a more lenient threshold than usual because of the large differences in degree distribution within the three networks. Nevertheless, we still were able to identify multiple hubs per network and extract biological functions for these hubs (Table 1). Of the three categories that the functions of the hubs can be classified into, the cell cycle/proliferation category is more abundant than the other two.

It is intriguing to note that most of the hubs of the three networks are known for being high level regulators for several biological processes, which supports the relevance of this network analysis. The hubs from the overlapping network, **Tr1&Tr2** are involved in diverse biological processes and signalling/cascading events. The expression of all the hubs in the **OnlyTr1** and the **Tr1&Tr2** networks is significantly different over time in comparison with their expression pattern in the control group. We speculate that the treatments directly or indirectly affect these hubs which then regulate genes to bring about the long term effects of the treatments. The hubs in the **OnlyTr2** network conspicuously do not have significant expression level differences over the time span of the experiment when compared to the control group. This suggests that the stress component of the treatment does not affect the animal in the long term as much as the antibiotic alone does.

### Biological relevance of the hubs

Among the hubs, there are two STAT genes, STAT3 and STAT1, in the **OnlyTr1** and **OnlyTr2** networks respectively. The STAT family of genes are well studied and have a role in signalling events but also act as transcription factors [75]. The STAT1, STAT2, STAT3, STAT4 genes, found to have significantly different temporal expression patterns in the treatments versus the control, are especially related to immune reactions [75–77]. This is in agreement with the results of our functional analysis of the networks. STAT3 and STAT1 are known to be up-regulated in cases of bacterial infection [75, 78–80]. In addition, expression of STAT1 has been reported to be reduced in the presence of antibiotics [76, 78, 80, 81]. The latter is reflected in our data by the temporal expression patterns shown in Fig. 3. Though the overall expression patterns of STAT1 among the three groups are similar, antibiotic treatment and the concomitant change in microbial communities, has a clear effect on STAT1 expression, especially at the first two time-points. Expression of STAT1 on day 8 in Tr2 is, however, quite high compared to the Ctrl, this could indicate that the stress applied at day 4 counteracts the effect of the antibiotic. STAT3 is known to be important for survival of embryos [75, 76, 82], it is important during the early stages of development but is not found to be essential in adult tissue [76]. This is reflected in the observed decreasing expression levels of STAT3 over the three time-points (Fig. 3). The observed contrasting expression pattern of the STAT1 and STAT 3 hubs, may be related to the previous observations that STAT1 and STAT3 counteract each other [80, 83].

### Data integration

With regard to the microbiota composition and diversity, this study revealed a more prominent effect of time than that of treatment. This is in accordance with previous observations that indicate that the diversity and composition of intestinal microbiota change considerably over time [84, 85]. As expected, the treatment effects are strongest at the first time point, taking into account the efficacy of the used antibiotic [25, 86]. As soon as the antibiotic wears off, the microbiota returns to the “normal” state. This is consistent with known literature about the resilience of microbial communities [87, 88].

Since the gene expression data as well as the microbiota data change with treatment and time, this presents an opportunity to correlate these different types of information and look into probable relationships and interactions in the jejunum between microbiota and gene expression of the cells in the mucosal layer. We assumed that the **OnlyTr1** and **Tr1&Tr2** networks represent the effect of the antibiotic on the cross-talk between microbiota and mucosal cells. The **OnlyTr2** correlation network could be interpreted as a representation of the cross-talk between the host and microbiota in response to stress, although we did not look at the effect of physical stress alone. The bacterial groups with the most gene connections are common to the three correlation networks. This indicates that the two treatments do not have drastic effects on the possible host microbiota interactions. The **OnlyTr2** correlation network has more bacterial groups that correlate with genes which suggests that the physical stress factor alters the cross-talk more than the antibiotic alone does. The number of known pathobionts increases in the latter network which could be an indication of a tendency towards pathology in stressful conditions.

### Crosstalk between host and microbe components

There are three bacterial groups that correlate with a high number of genes in all the three networks: *Eubacterium*; *Ruminococcus bromii*; and *Faecalibacterium. Eubacterium* is a genus with very diverse species. *Ruminococcus bromii* is a species that has been extensively studied for its ability to breakdown resistant starch [89] to produce acetate on which other bacterial groups can survive. Another beneficial bacterial group is *Faecalibacterium* which is a relatively new genus with only one species documented so far, *F. parusnitzii* and it has been reported to be extremely beneficial to the host. *Ruminococcus bromii*; and *Faecalibacterium* are reported to be most abundant in the anaerobic environment of the colon [90, 91]. Nevertheless, there is evidence that the small intestine is populated with quite a few of such fermenters [92]. In our analysis the abundance of these bacterial groups in the jejunum correlates to the expression level of a considerable number of genes. This suggests that they are major players in the cross-talk in the jejunum. *Lawsonia intracellularis* and species of *Campylobacter* are found to be correlated with several genes. These groups are known mammalian pathogens of the small intestine. It could be speculated that, in these healthy animals, they are involved in balancing between immune tolerance and immune responses.

### Understanding the long lasting changes in the host

The results of the regression analysis on gene expression alone show that there are differences between the three treatment groups for a long time after the perturbation. These differences are reflected in immune functions and cell proliferation related functions. It has been suggested that stress and antibiotics compromise the immune system, but in different ways [27, 35, 38]. The action of antibiotics could change the delicately balanced signalling between microbiota and the intestinal cells. This change during early stages of development is expected to influence the development and programming of the immune system with consequences for the later life functioning of the immune system. It has been proposed that macrolides (the type of antibiotic given in this experiment) work by recruiting immune cells to carry the antibiotic to the afflicted tissue [93, 94]. This mode of action will bring about temporary changes in the immune system but cannot explain the observation as described here. Stress is known to cause structural changes in the intestinal tissue [38–40] and may affect the microbial populations [34].

The above described changes are not expected to last over a long period of time; intestinal cells have a large turnover [95, 96] almost every 4 to 5 days. Yet we find changes that persist for 51 and even 172 days after the perturbation of the gut system. This suggests that the programming and development of the immune system occurred differently between the different treatment groups. Since the microbial composition at day 8 significantly differed between the treatments groups we believe that the difference in immune programming and development is due to differences in the early life crosstalk in the gut between microbes and host. This line of thinking is agreement with the conclusions in several other recent studies in this area [2, 97]. It is also in line with our own observation that the microbiota composition returns to the normal state briefly after the perturbation. Memory cells of the mucosal immune system could play an important role in the programming of the mucosal immune system [30, 67, 98]. Furthermore even though stress was not expected to play a very prominent role in changing the system in the long term, we see that in some instances, it counteracts the effects of the antibiotic. The mechanism behind this is yet to be fully understood.

Pigs are regarded as a useful model for research into modulation of the human gut, especially with regard to microbiota-host immune interactions [99]. This experiment was designed to disturb the pattern of early colonization of the gut by microbiota with known effects later in life [25]. The factors used for perturbation were, treatment with an antibiotic or an antibiotic with stress at day 4 after birth. Both human neonates as well as piglets in intensive husbandry systems may be exposed to such factors. Therefore our results may be relevant for both humans and piglets.

## Conclusions

We used an early life intestinal perturbation in piglets and demonstrate long lasting effects, as measured on the intestinal gene expression level. We provide substantial evidence that several biological processes of the gut mucosal tissue are in a different state between the experimental groups over a long period of time. However, the regression analysis did not identify significant differences among the temporal patterns of the bacterial species, and the treatments do not seem to affect long term changes in the microbiota. We conclude that the difference in immune programming and development is due to differences in the early life crosstalk in the gut between microbes and host, resulting from the perturbations. Since we identified potential high-level regulators of long term changes in the gut, we are one step closer to identifying the underlying mechanisms. Although the treatments did not lead to phenotypical manifestations, like weight differences between the animals, the exposure of pigs to stressors like pathogen challenges could bring out immune variations between the groups. Studying these subtle differences may help to develop strategies to modulate the process of immune development and programming. The results of this paper are supportive for the recent notion that antibiotics should be used more carefully in neonatal humans and animals.

### Availability of data

The data used in this research article is available on Gene Expression Omnibus with the accession number GSE53170, through this link http://www.ncbi.nlm.nih.gov/geo/query/acc.cgi?acc=GSE53170.

### Ethics statement

The experiment was approved by the institutional animal experiment committee “Dier Experimenten Commissie (DEC) Lelystad” (2011077.b), in accordance with the Dutch regulations on animal experiments.

## Additional files

Additional file 1: Figure S1. Experimental Design. The figure shows the timeline of the experiment, and each line shows a different group of animals. The red line represents the control group, the blue line the Antibiotics alone (Tr1) and the green line the group given Antibiotics and Stress (Tr2). The pink block represents the intervention of the groups and the light blue boxes represent times of sampling. The boxes at the bottom represent the distribution of the groups among the sows, the pigs were housed in the same manner after weaning. Additional file 2: Figure S2. Microarray Analysis. Workflow of the microarray analysis with the specific details. Additional file 3: Figure S3. Types of Differences in Time Profiles. Each graph depicts the temporal expression pattern of a single gene. These temporal changes are shown under three different conditions: Ctrl (red line), Tr1 (green line), and Tr2 (blue line). The x-axis indicates the time in days, the y-axis has expression values scaled such that the average expression of each gene is 0 and the standard deviation is 1. Additional file 4: Figure S4. Expression of FI network hubs. Expression profiles of 17 hubs of the three FI networks. Each graph depicts the temporal expression pattern of a single gene. These temporal changes are shown under three different conditions: Ctrl (red line), Tr1 (green line), and Tr2 (blue line). The x-axis indicates the time in days, the y-axis has expression values scaled such that the average expression of each gene is 0 and the standard deviation is 1. Additional file 5: Figure S5. Original Tr1&Tr2 FI network. Network formed with genes that have significantly different time profiles between both treatments vs the control group. Additional file 6: Figure S6. Temporal changes in composition of bacterial groups used in the correlation networks. Each graph represents the compositional changes of selected bacterial groups over time. The bacterial groups were chosen based on an ANOVA analysis. The time in days is on the x-axis, the log values of the contribution of the bacterial groups is on the y-axis. Each of the 46 graphs have a different scale on the y-axis due to the extreme differences between contributions of each of the bacterial groups. Additional file 7: Figure S7. OnlyTr1 Reactome FI network. Additional file 8: Figure S8. Tr1&Tr2 Reactome FI network. Additional file 9: Figure S9. OnlyTr2 Reactome FI network. Additional file 10: Figure S10. OnlyTr1 correlation network. Additional file 11: Figure S11. Tr1&Tr2 correlation network. Additional file 12: Figure S12. OnlyTr2 correlation network. Additional file 13:
**List of significant genes in different conditions.**
Additional file 14:
**Information on the Reactome FI networks.**
Additional file 15:
**Information on the correlation networks.**

## Abbreviations

GI: Gastrointestinal
Tr1: Treatment group 1
Tr2: Treatment group 2
Ctrl: Control group
DEC: Dier experimenten commissie
PITChip: Pig intestinal tract chip
GEO: Gene Expression Omnibus
LIMMA: LInear models for microarray data
maSigPro: Microarray significant profiles
GO: Gene ontology
FI: Functional interactions
MySQL: My structured query language
ANOVA: Analysis of variance
