# Supplementary figures and images for: Network analysis of temporal functionalities of the gut induced by perturbations in new-born piglets
Source: BMC Genomics. 2015 Jul 29;16(1):556. doi: 10.1186/s12864-015-1733-8 (PMC4518884; doi:10.1186/s12864-015-1733-8)

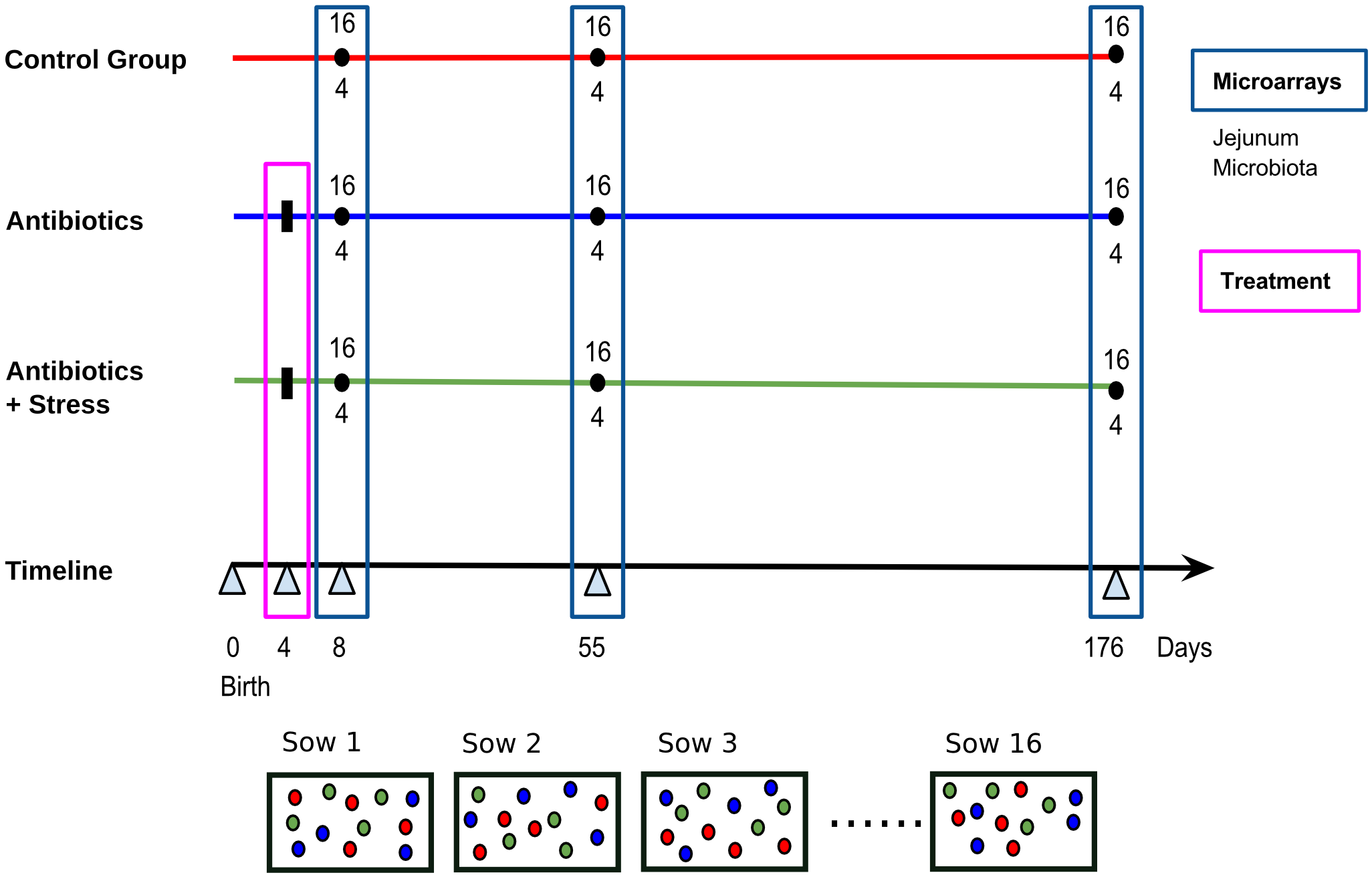

Supplement: Additional file 1: Figure S1. — Experimental Design. The figure shows the timeline of the experiment, and each line shows a different group of animals. The red line represents the control group, the blue line the Antibiotics alone (Tr1) and the green line the group given Antibiotics and Stress (Tr2). The pink block represents the intervention of the groups and the light blue boxes represent times of sampling. The boxes at the bottom represent the distribution of the groups among the sows, the pigs were housed in the same manner after weaning. [file 12864_2015_1733_MOESM1_ESM.png]

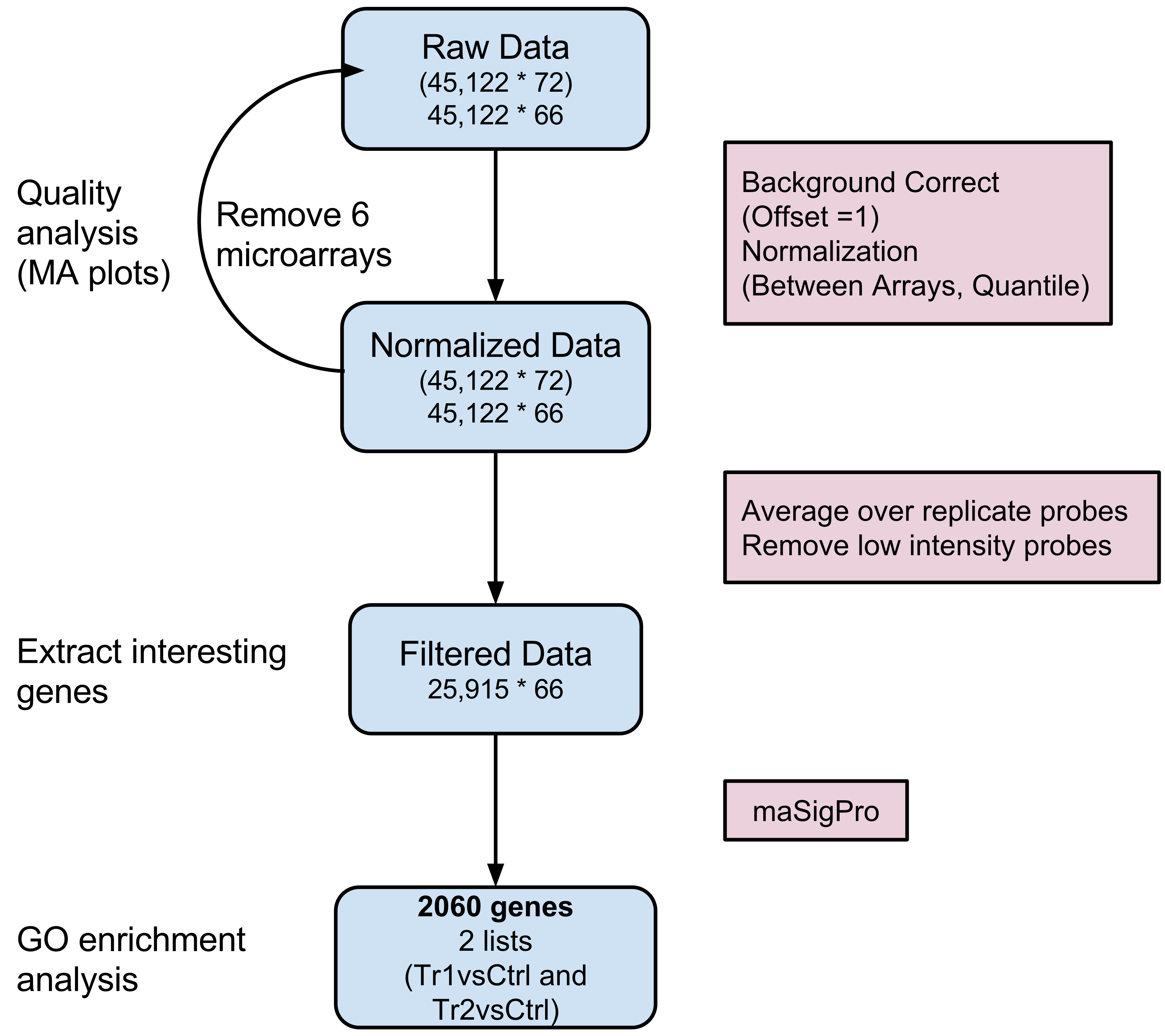

Supplement: Additional file 2: Figure S2. — Microarray Analysis. Workflow of the microarray analysis with the specific details. [file 12864_2015_1733_MOESM2_ESM.png]

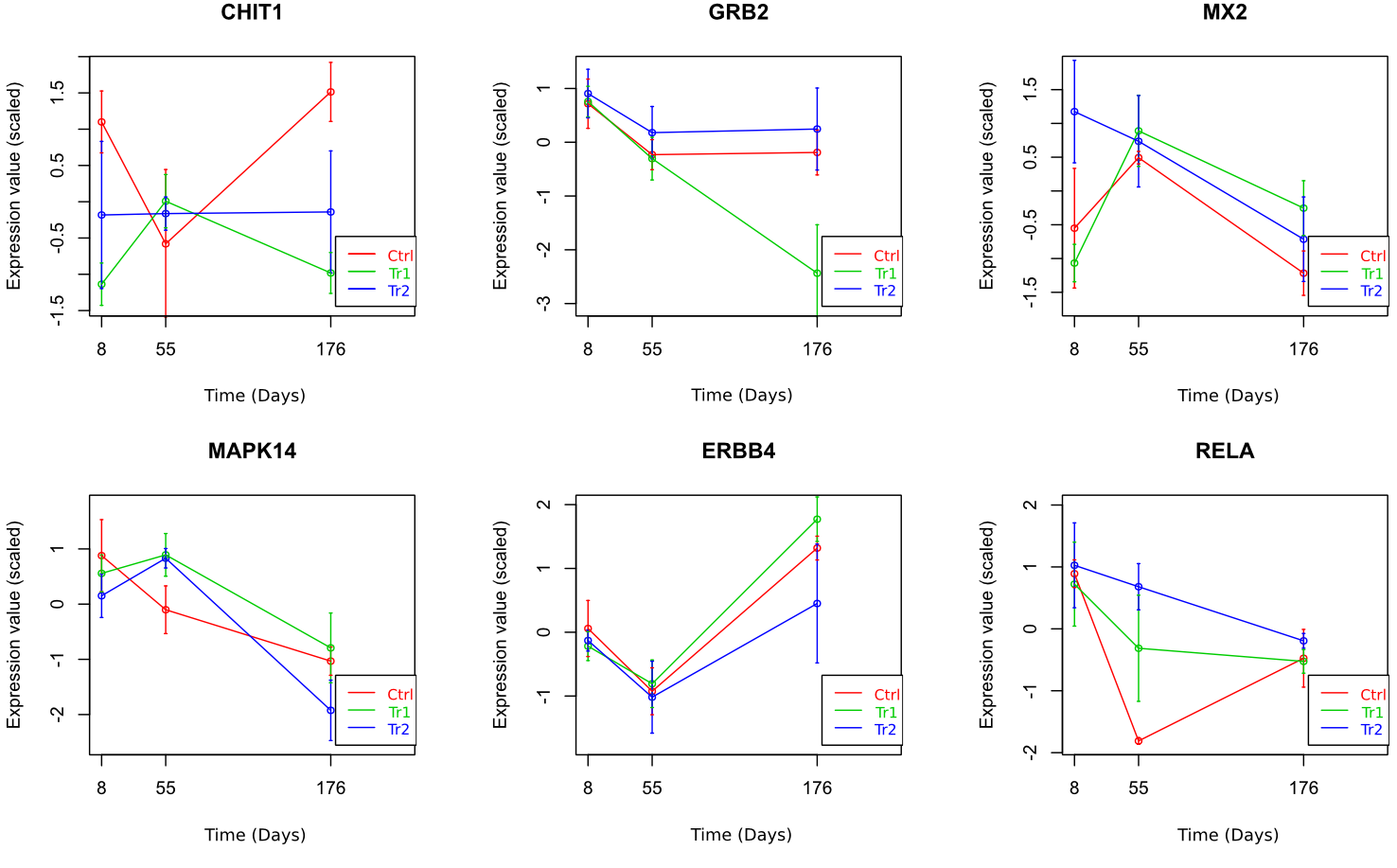

Supplement: Additional file 3: Figure S3. — Types of Differences in Time Profiles. Each graph depicts the temporal expression pattern of a single gene. These temporal changes are shown under three different conditions: Ctrl (red line), Tr1 (green line), and Tr2 (blue line). The x-axis indicates the time in days, the y-axis has expression values scaled such that the average expression of each gene is 0 and the standard deviation is 1. [file 12864_2015_1733_MOESM3_ESM.png]

**GRB2**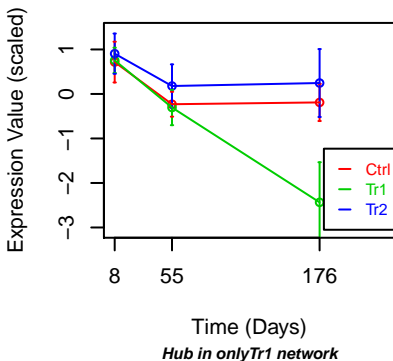**STAT3**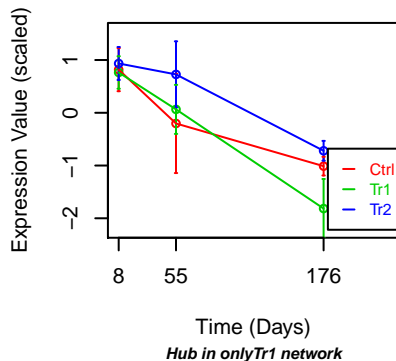**CDC42**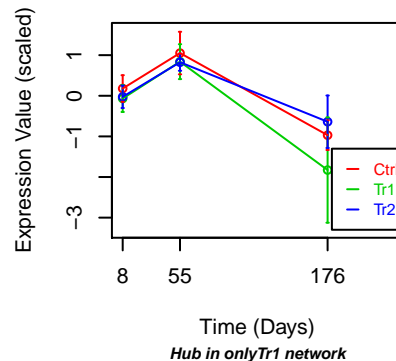**CAV1**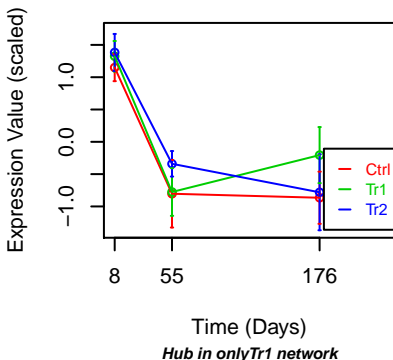**FOS**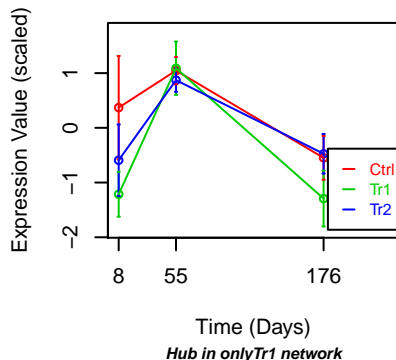**MYC**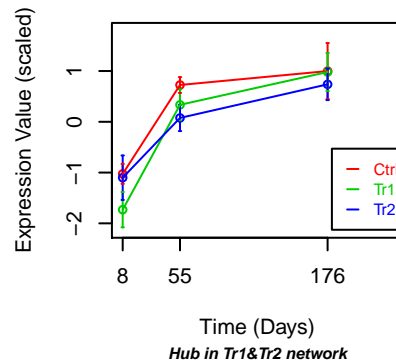**MAPK14**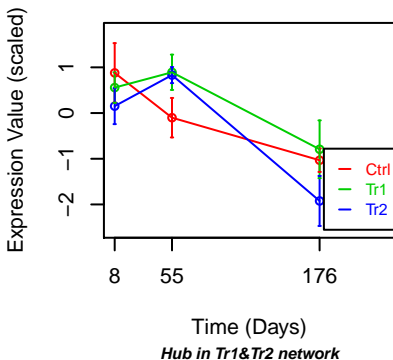**RELA**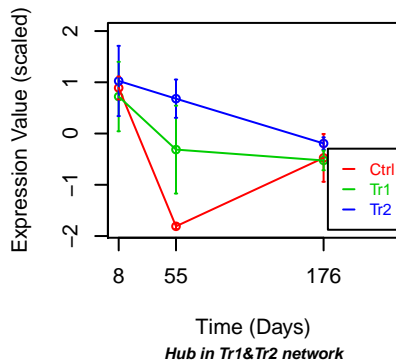**UBE2D2**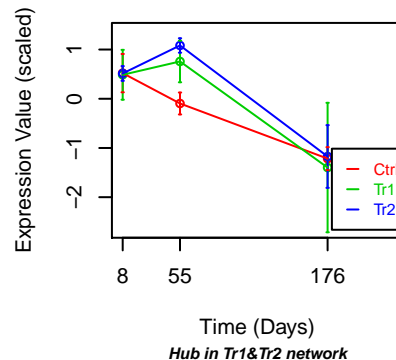

### ITCH

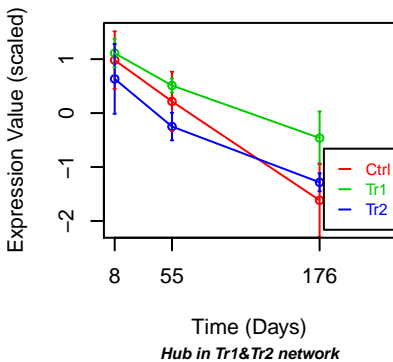

### IKBKG

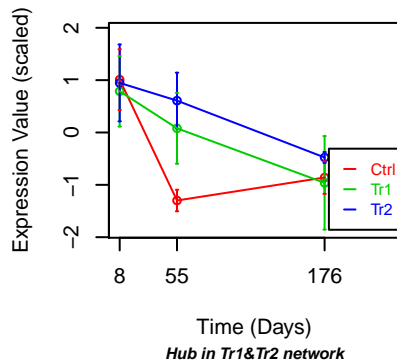

### SP1

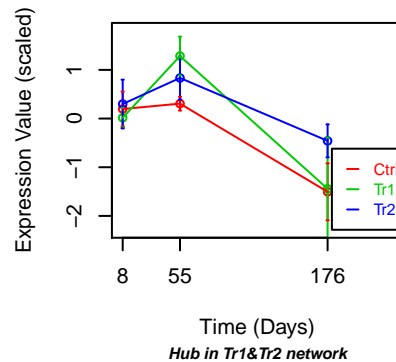

### RHOA

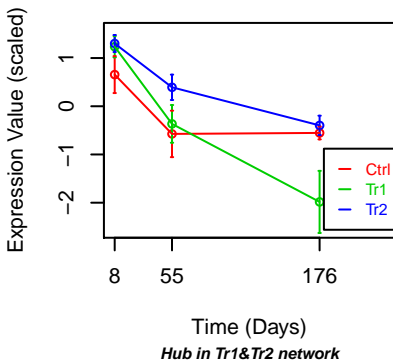

### RPS3

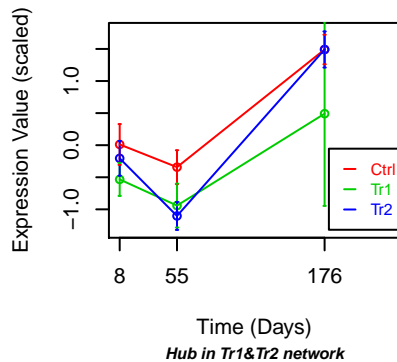

### FBXW7

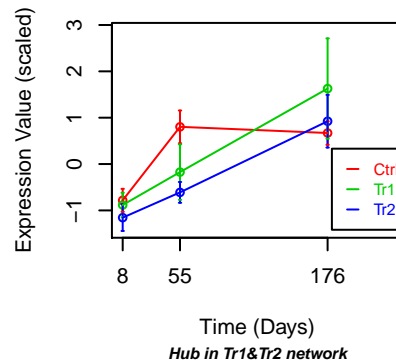

### UBA52

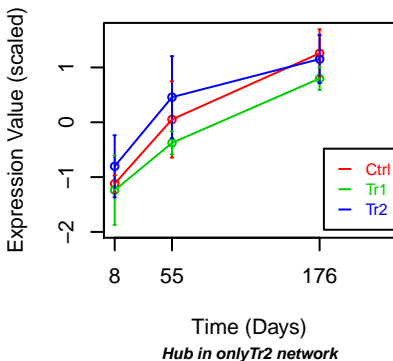

### STAT1

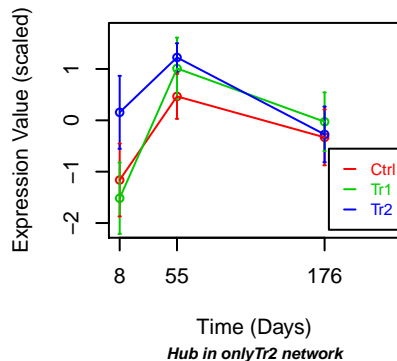

Supplement: Additional file 4: Figure S4. — Expression of FI network hubs. Expression profiles of 17 hubs of the three FI networks. Each graph depicts the temporal expression pattern of a single gene. These temporal changes are shown under three different conditions: Ctrl (red line), Tr1 (green line), and Tr2 (blue line). The x-axis indicates the time in days, the y-axis has expression values scaled such that the average expression of each gene is 0 and the standard deviation is 1. [file 12864_2015_1733_MOESM4_ESM.pdf]

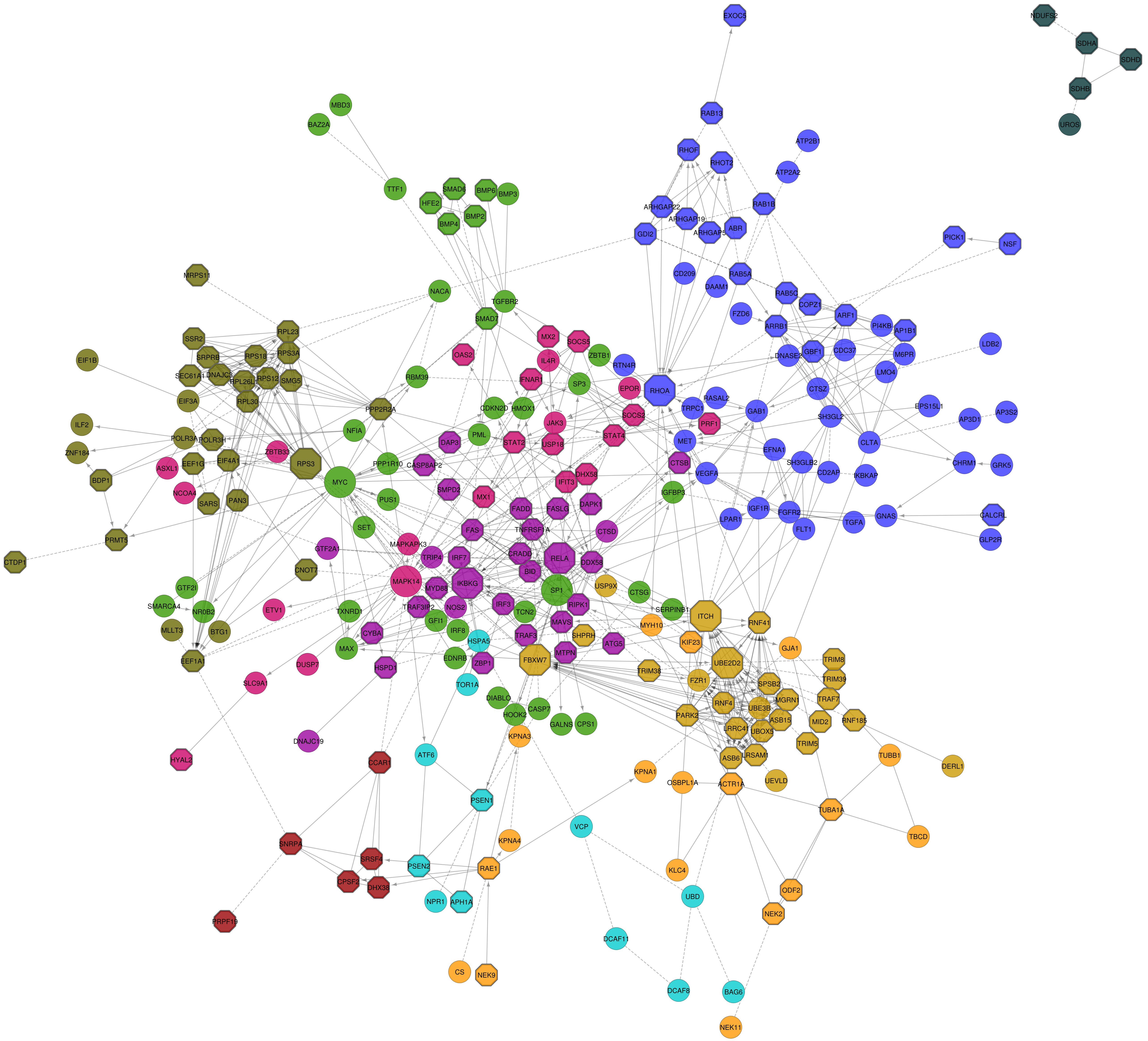

Supplement: Additional file 5: Figure S5. — Original Tr1&Tr2 FI network. Network formed with genes that have significantly different time profiles between both treatments vs the control group. [file 12864_2015_1733_MOESM5_ESM.png]

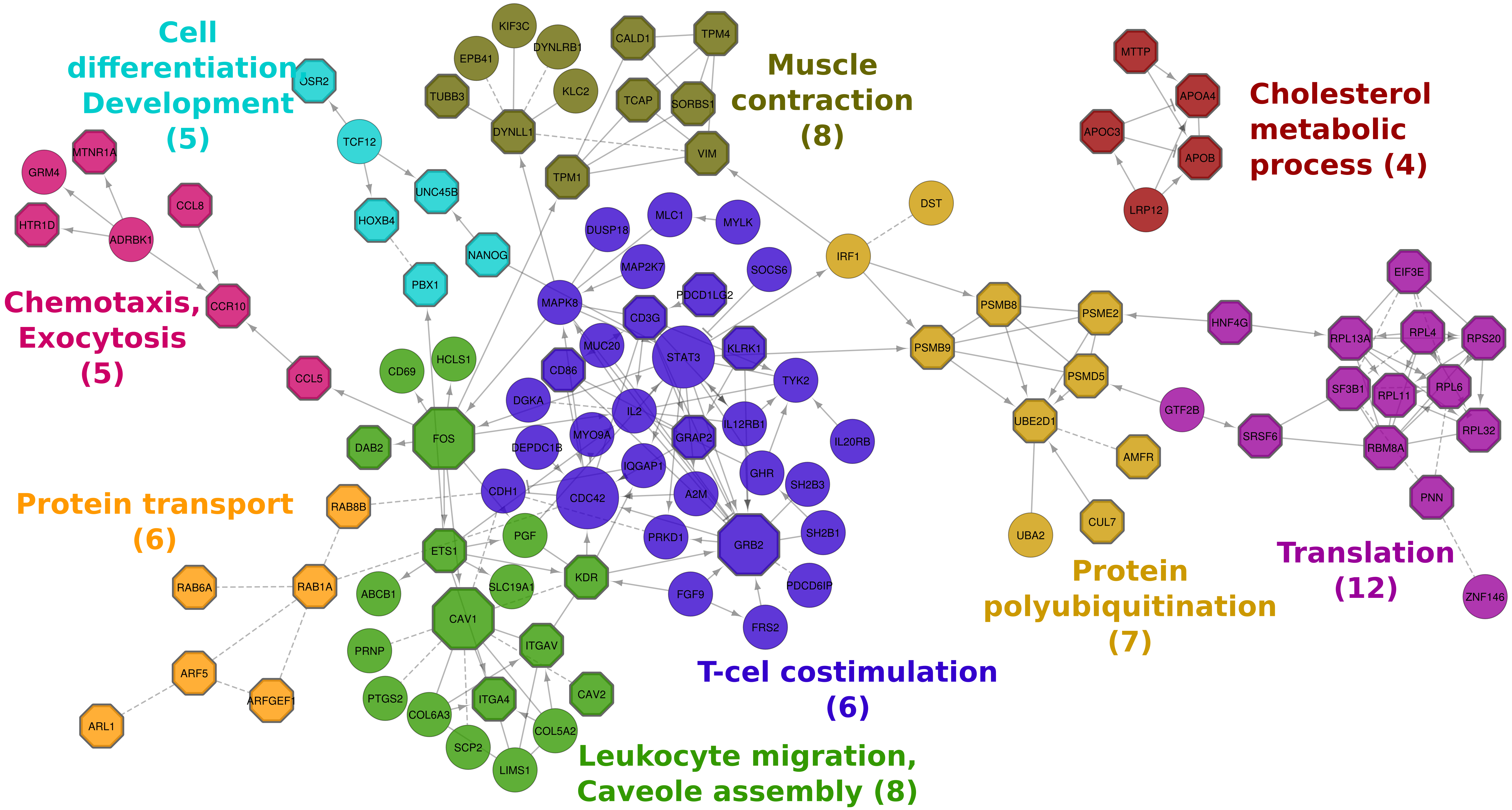

Supplement: Additional file 7: Figure S7. — OnlyTr1 Reactome FI network. [file 12864_2015_1733_MOESM7_ESM.png]

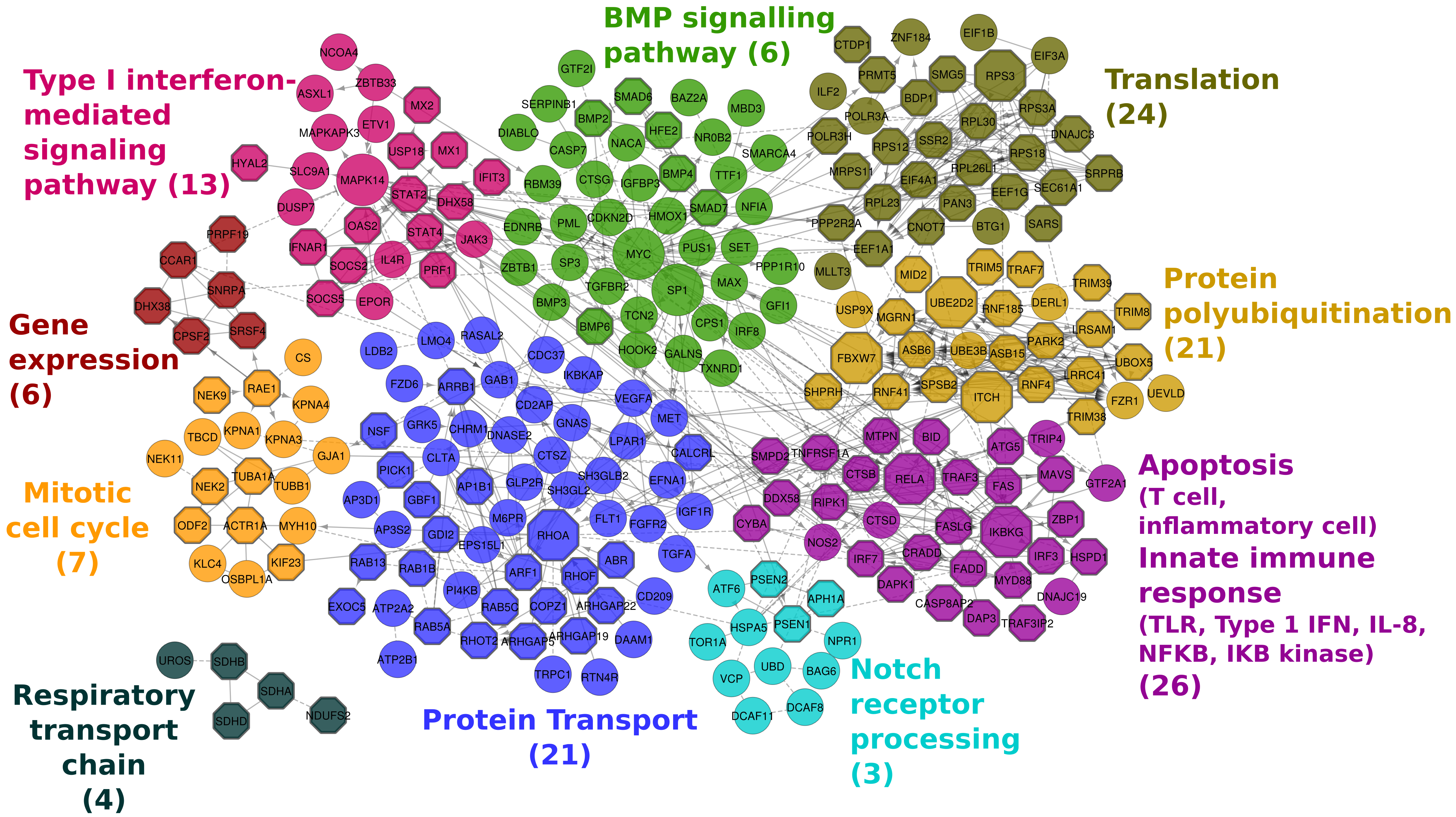

Supplement: Additional file 8: Figure S8. — Tr1&Tr2 Reactome FI network. [file 12864_2015_1733_MOESM8_ESM.png]

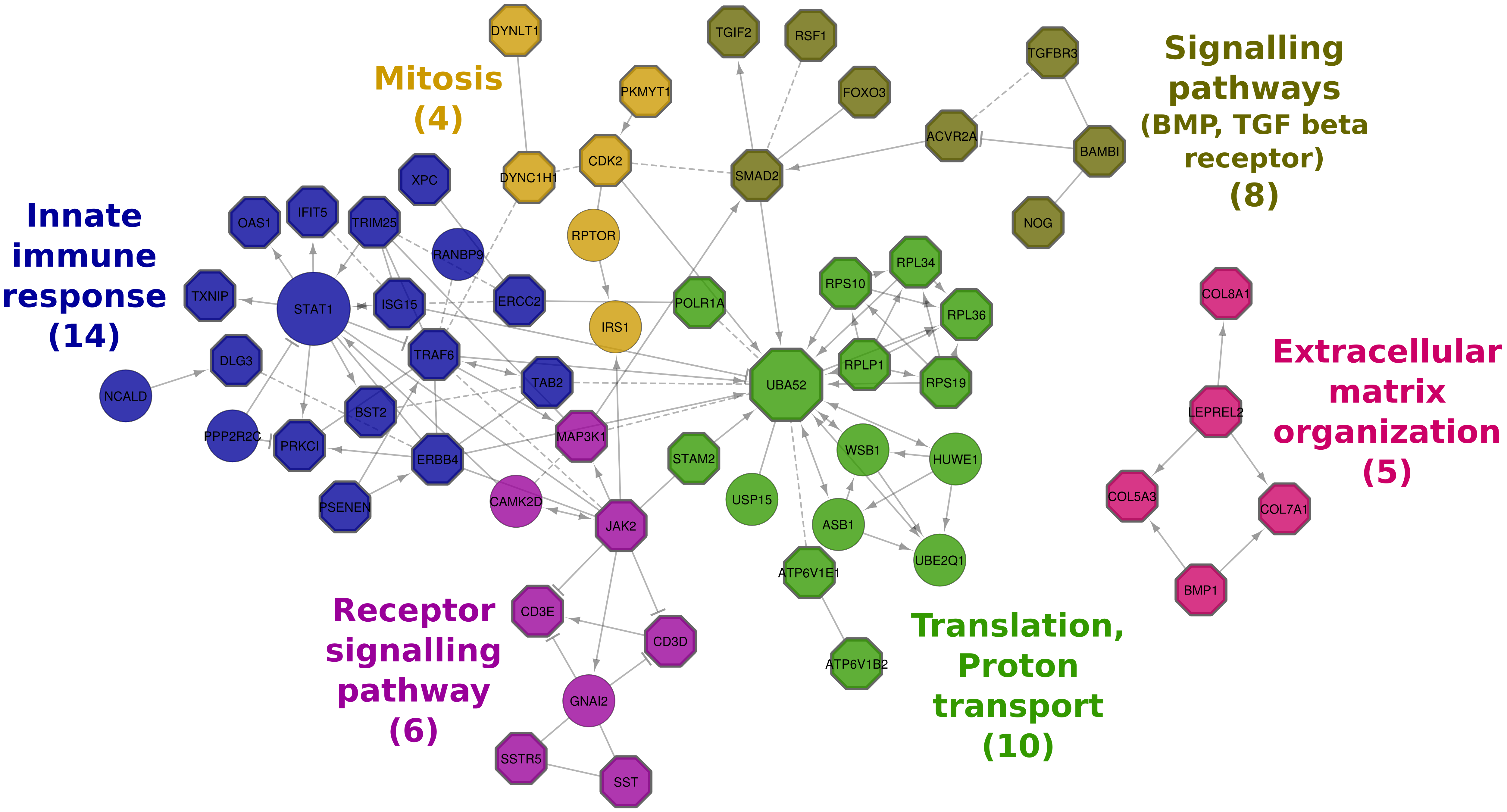

Supplement: Additional file 9: Figure S9. — OnlyTr2 Reactome FI network. [file 12864_2015_1733_MOESM9_ESM.png]

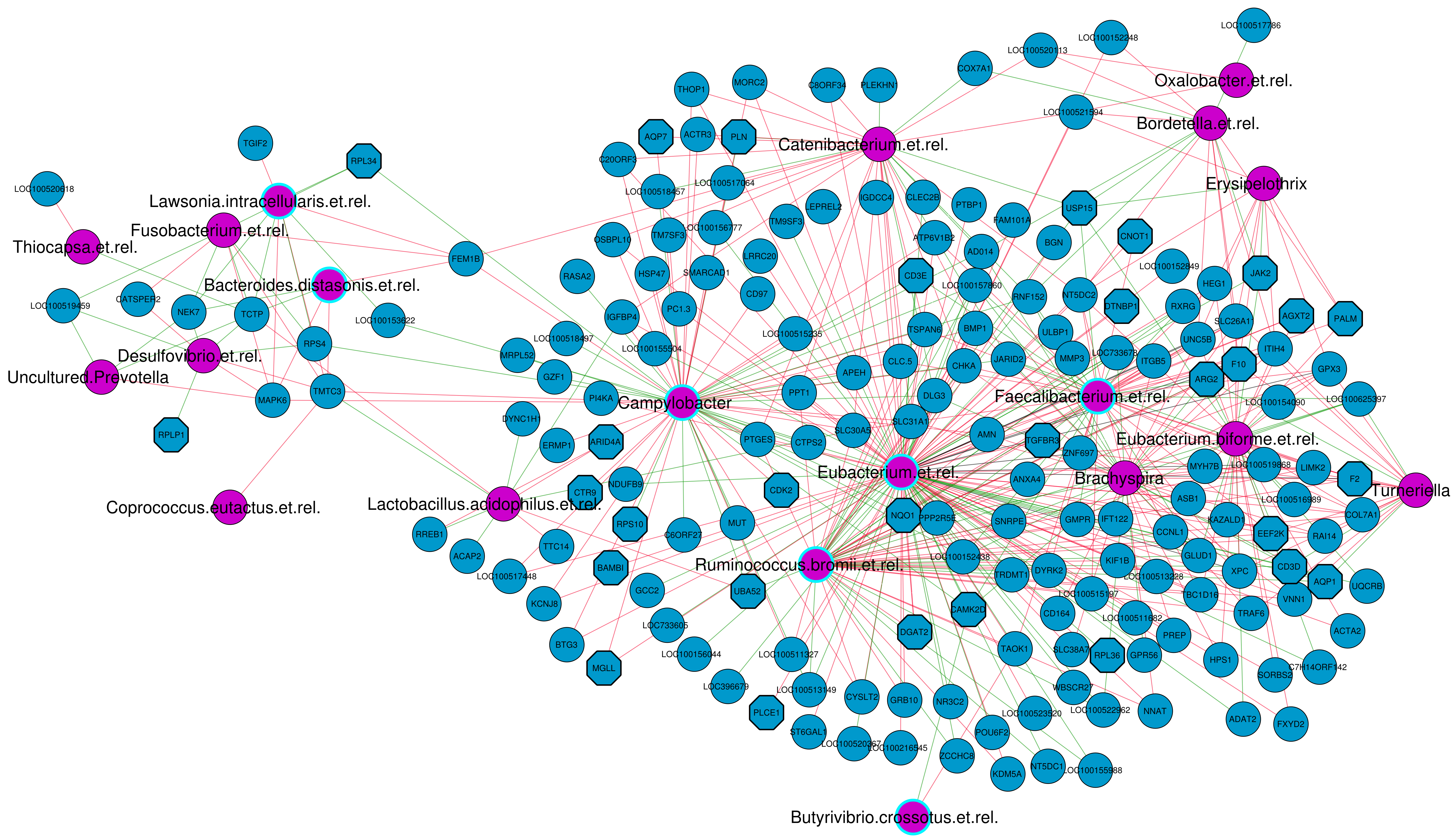

Supplement: Additional file 10: Figure S10. — OnlyTr1 correlation network. [file 12864_2015_1733_MOESM10_ESM.png]

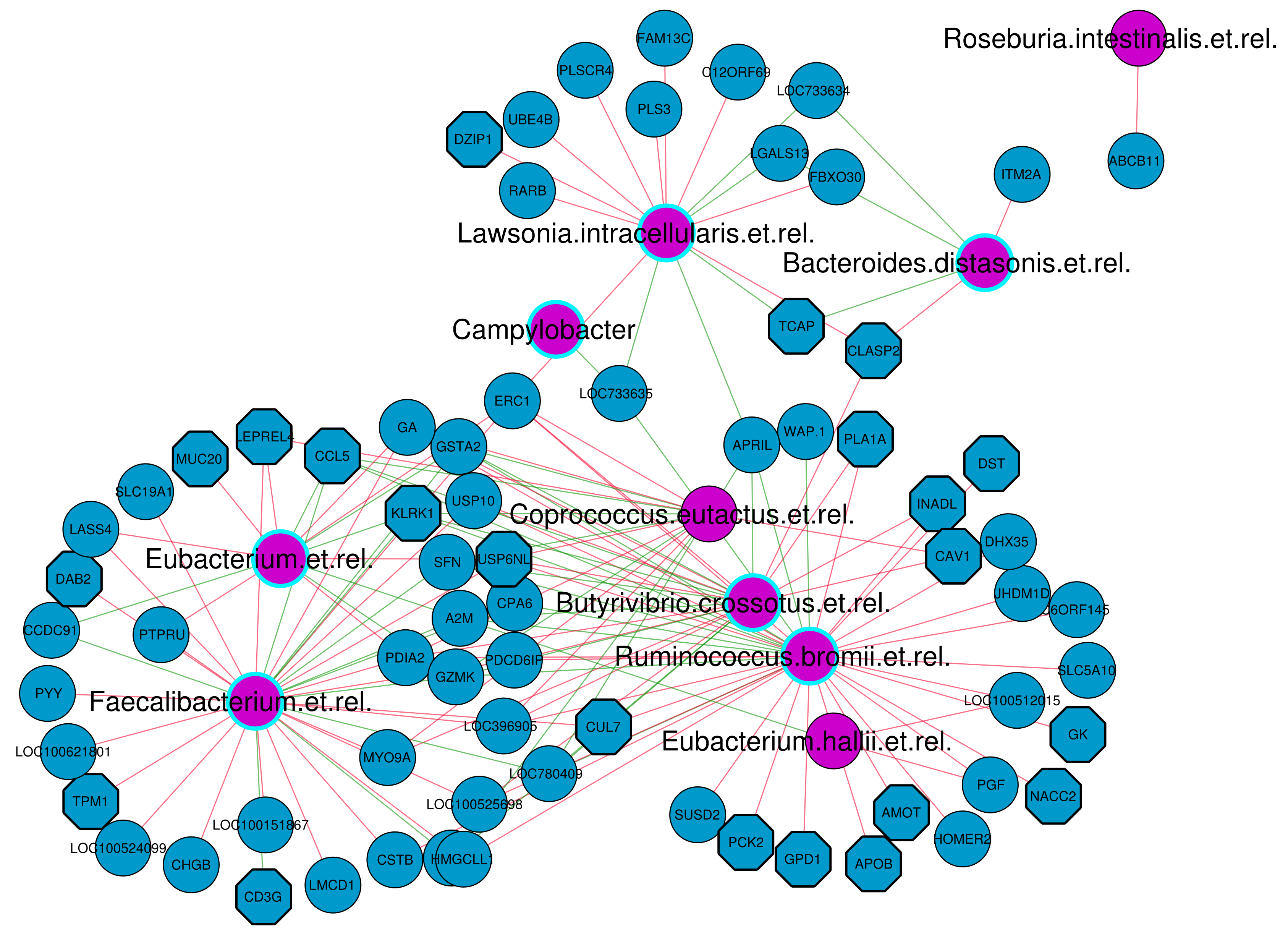

Supplement: Additional file 11: Figure S11. — Tr1&Tr2 correlation network. [file 12864_2015_1733_MOESM11_ESM.png]

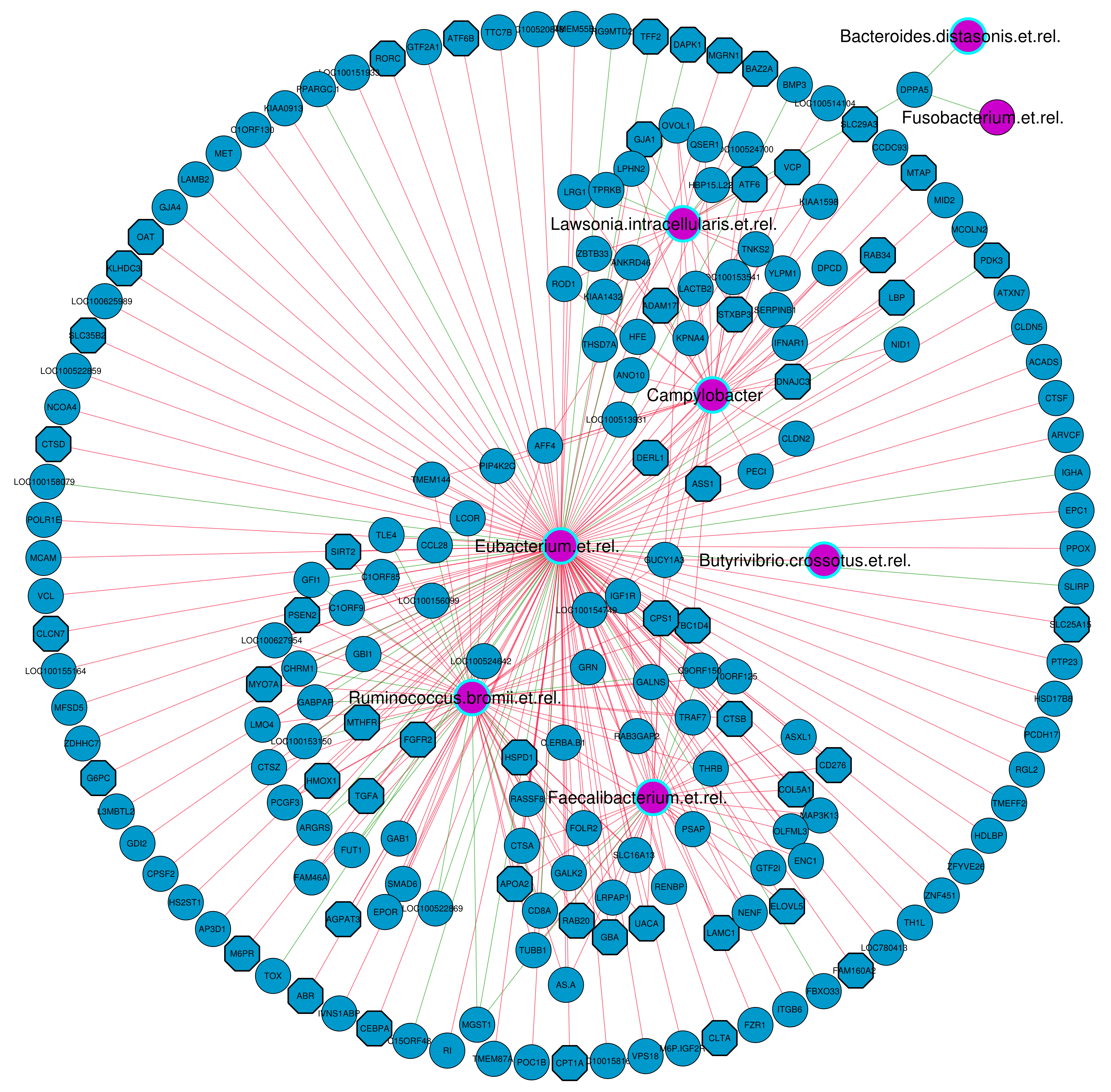

Supplement: Additional file 12: Figure S12. — OnlyTr2 correlation network. [file 12864_2015_1733_MOESM12_ESM.png]
