# Supplementary material for: Network analysis of temporal functionalities of the gut induced by perturbations in new-born piglets
Source: BMC Genomics. 2015 Jul 29;16(1):556. doi: 10.1186/s12864-015-1733-8 (PMC4518884; doi:10.1186/s12864-015-1733-8)
Supplement: Additional file 6: Figure S6. — Temporal changes in composition of bacterial groups used in the correlation networks. Each graph represents the compositional changes of selected bacterial groups over time. The bacterial groups were chosen based on an ANOVA analysis. The time in days is on the x-axis, the log values of the contribution of the bacterial groups is on the y-axis. Each of the 46 graphs have a different scale on the y-axis due to the extreme differences between contributions of each of the bacterial groups. [file 12864_2015_1733_MOESM6_ESM.pdf]

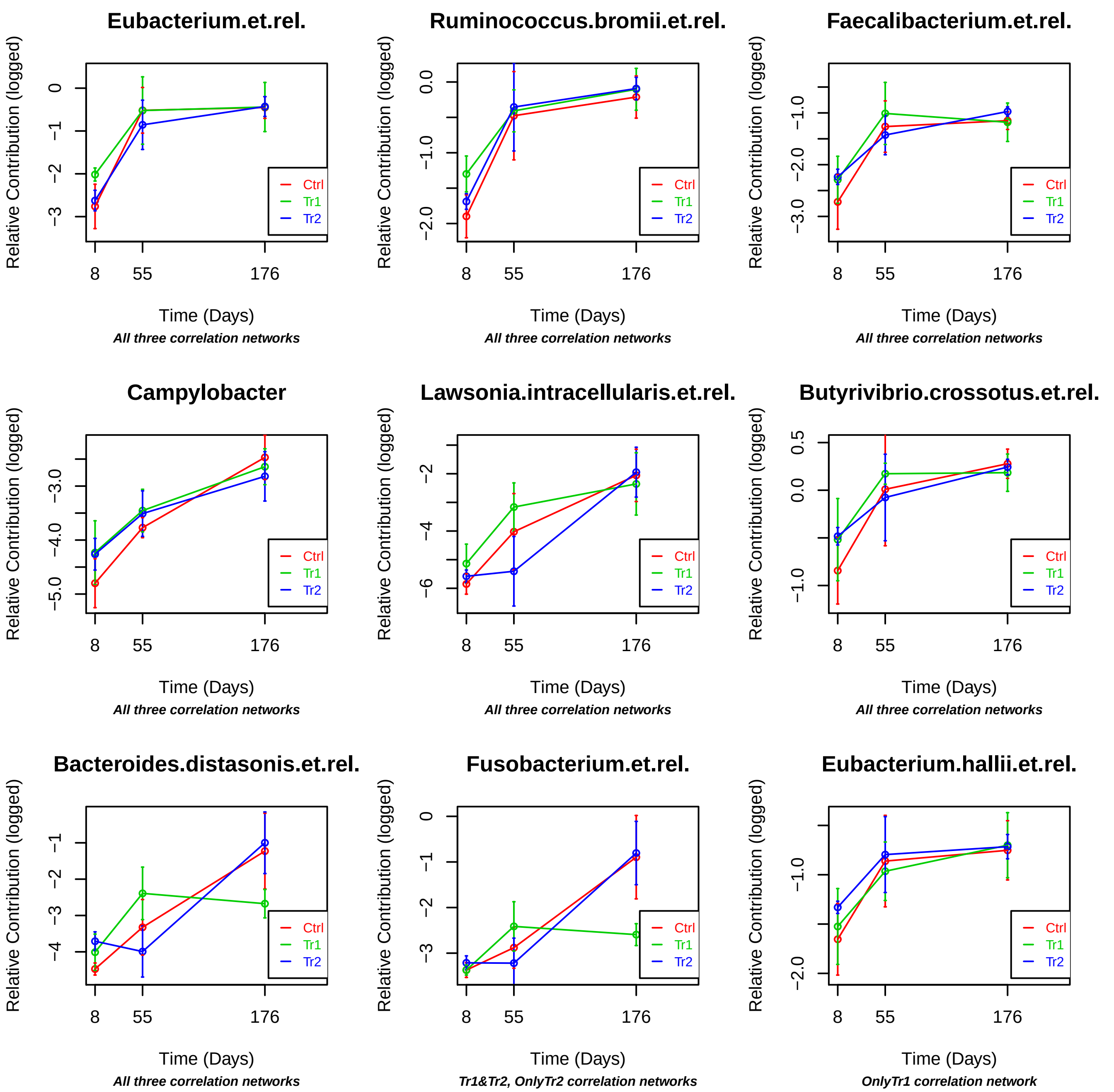
Eubacterium.et.rel.
Relative Contribution (logged)
0
−1
−2
−3
8
55
176
Time (Days)
All three correlation networks
Ctrl
Tr1
Tr2
Ruminococcus.bromii.et.rel.
Relative Contribution (logged)
0.0
−1.0
−2.0
8
55
176
Time (Days)
All three correlation networks
Ctrl
Tr1
Tr2
Faecalibacterium.et.rel.
Relative Contribution (logged)
−1.0
−2.0
−3.0
8
55
176
Time (Days)
All three correlation networks
Ctrl
Tr1
Tr2
Campylobacter
Relative Contribution (logged)
−3.0
−4.0
−5.0
8
55
176
Time (Days)
All three correlation networks
Ctrl
Tr1
Tr2
Lawsonia.intracellularis.et.rel.
Relative Contribution (logged)
−2
−4
−6
8
55
176
Time (Days)
All three correlation networks
Ctrl
Tr1
Tr2
Butyrivibrio.crossotus.et.rel.
Relative Contribution (logged)
0.5
0.0
−1.0
8
55
176
Time (Days)
All three correlation networks
Ctrl
Tr1
Tr2
Bacteroides.distasonis.et.rel.
Relative Contribution (logged)
−1
−2
−3
−4
8
55
176
Time (Days)
All three correlation networks
Ctrl
Tr1
Tr2
Fusobacterium.et.rel.
Relative Contribution (logged)
0
−1
−2
−3
8
55
176
Time (Days)
Tr1&Tr2, OnlyTr2 correlation networks
Ctrl
Tr1
Tr2
Eubacterium.hallii.et.rel.
Relative Contribution (logged)
−1.0
−2.0
8
55
176
Time (Days)
OnlyTr1 correlation network
Ctrl
Tr1
Tr2

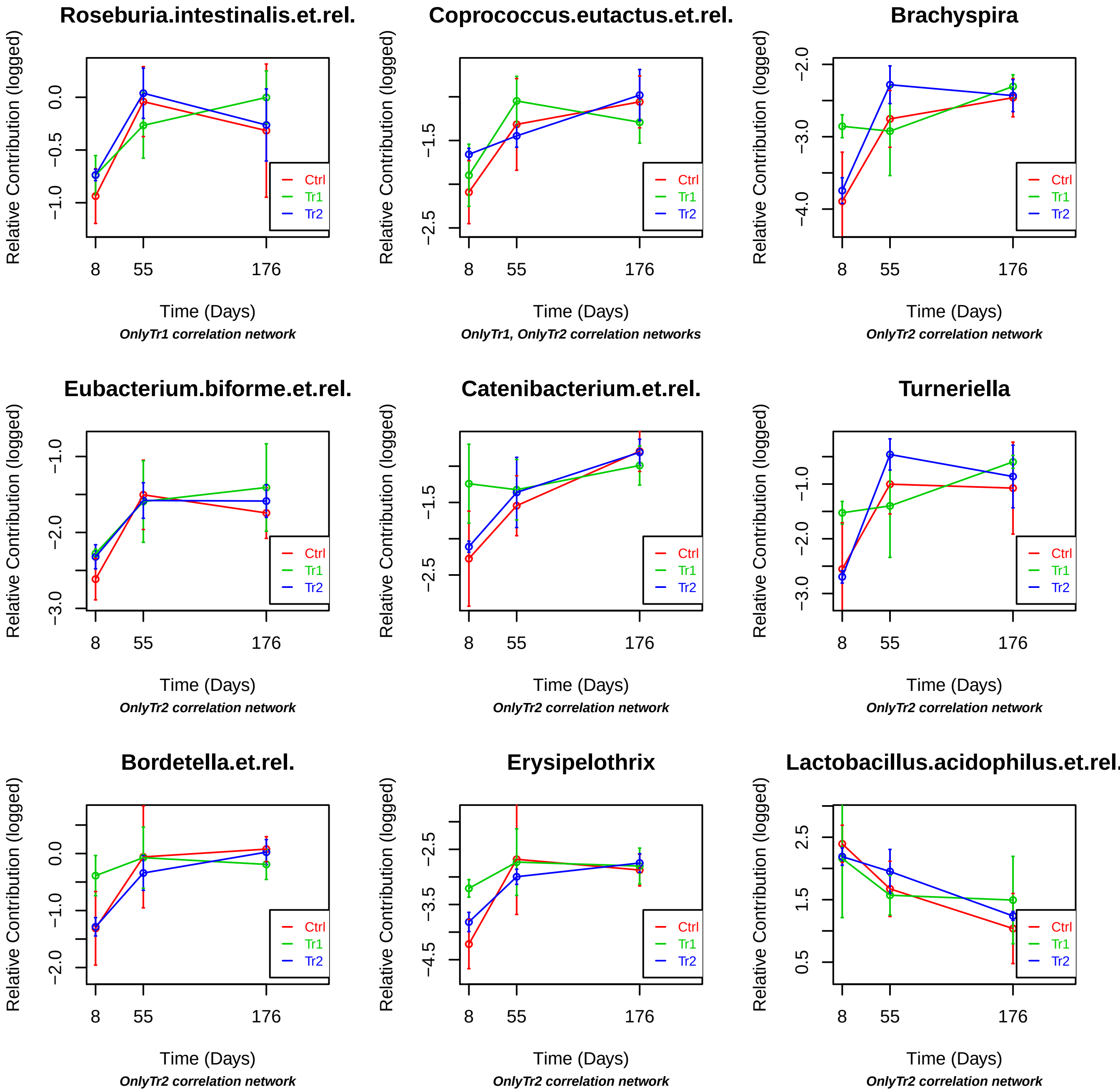

Roseburia.intestinalis.et.rel.
Relative Contribution (logged)
0.0
−0.5
−1.0
8
55
176
Time (Days)
OnlyTr1 correlation network
Ctrl
Tr1
Tr2
Coprococcus.eutactus.et.rel.
Relative Contribution (logged)
−1.5
−2.5
8
55
176
Time (Days)
OnlyTr1, OnlyTr2 correlation networks
Ctrl
Tr1
Tr2
Brachyspira
Relative Contribution (logged)
−2.0
−3.0
−4.0
8
55
176
Time (Days)
OnlyTr2 correlation network
Ctrl
Tr1
Tr2
Eubacterium.biforme.et.rel.
Relative Contribution (logged)
−1.0
−2.0
−3.0
8
55
176
Time (Days)
OnlyTr2 correlation network
Ctrl
Tr1
Tr2
Catenibacterium.et.rel.
Relative Contribution (logged)
−1.5
−2.5
8
55
176
Time (Days)
OnlyTr2 correlation network
Ctrl
Tr1
Tr2
Turneriella
Relative Contribution (logged)
−1.0
−2.0
−3.0
8
55
176
Time (Days)
OnlyTr2 correlation network
Ctrl
Tr1
Tr2
Bordetella.et.rel.
Relative Contribution (logged)
0.0
−1.0
−2.0
8
55
176
Time (Days)
OnlyTr2 correlation network
Ctrl
Tr1
Tr2
Erysipelothrix
Relative Contribution (logged)
−2.5
−3.5
−4.5
8
55
176
Time (Days)
OnlyTr2 correlation network
Ctrl
Tr1
Tr2
Lactobacillus.acidophilus.et.rel.
Relative Contribution (logged)
2.5
1.5
0.5
8
55
176
Time (Days)
OnlyTr2 correlation network
Ctrl
Tr1
Tr2

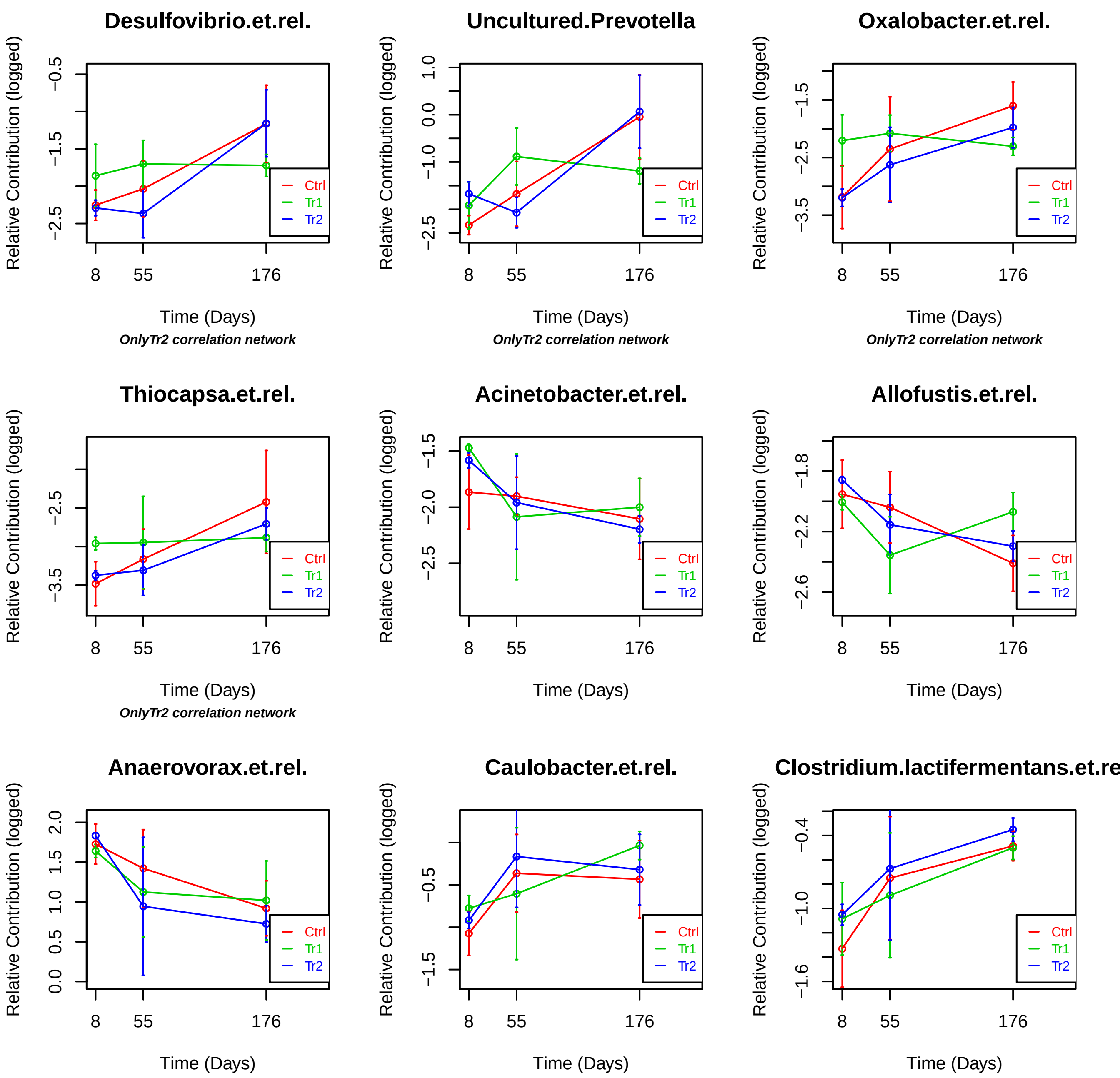
Desulfovibrio.et.rel.
Relative Contribution (logged)
−0.5
−1.5
−2.5
8
55
176
Time (Days)
OnlyTr2 correlation network
Ctrl
Tr1
Tr2
Uncultured.Prevotella
Relative Contribution (logged)
1.0
0.0
−1.0
−2.5
8
55
176
Time (Days)
OnlyTr2 correlation network
Ctrl
Tr1
Tr2
Oxalobacter.et.rel.
Relative Contribution (logged)
−1.5
−2.5
−3.5
8
55
176
Time (Days)
OnlyTr2 correlation network
Ctrl
Tr1
Tr2
Thiocapsa.et.rel.
Relative Contribution (logged)
−2.5
−3.5
8
55
176
Time (Days)
OnlyTr2 correlation network
Ctrl
Tr1
Tr2
Acinetobacter.et.rel.
Relative Contribution (logged)
−1.5
−2.0
−2.5
8
55
176
Time (Days)
Ctrl
Tr1
Tr2
Allofustis.et.rel.
Relative Contribution (logged)
−1.8
−2.2
−2.6
8
55
176
Time (Days)
Ctrl
Tr1
Tr2
Anaerovorax.et.rel.
Relative Contribution (logged)
2.0
1.5
1.0
0.5
0.0
8
55
176
Time (Days)
Ctrl
Tr1
Tr2
Caulobacter.et.rel.
Relative Contribution (logged)
−0.5
−1.5
8
55
176
Time (Days)
Ctrl
Tr1
Tr2
Clostridium.lactifermentans.et.re
Relative Contribution (logged)
−0.4
−1.0
−1.6
8
55
176
Time (Days)
Ctrl
Tr1
Tr2

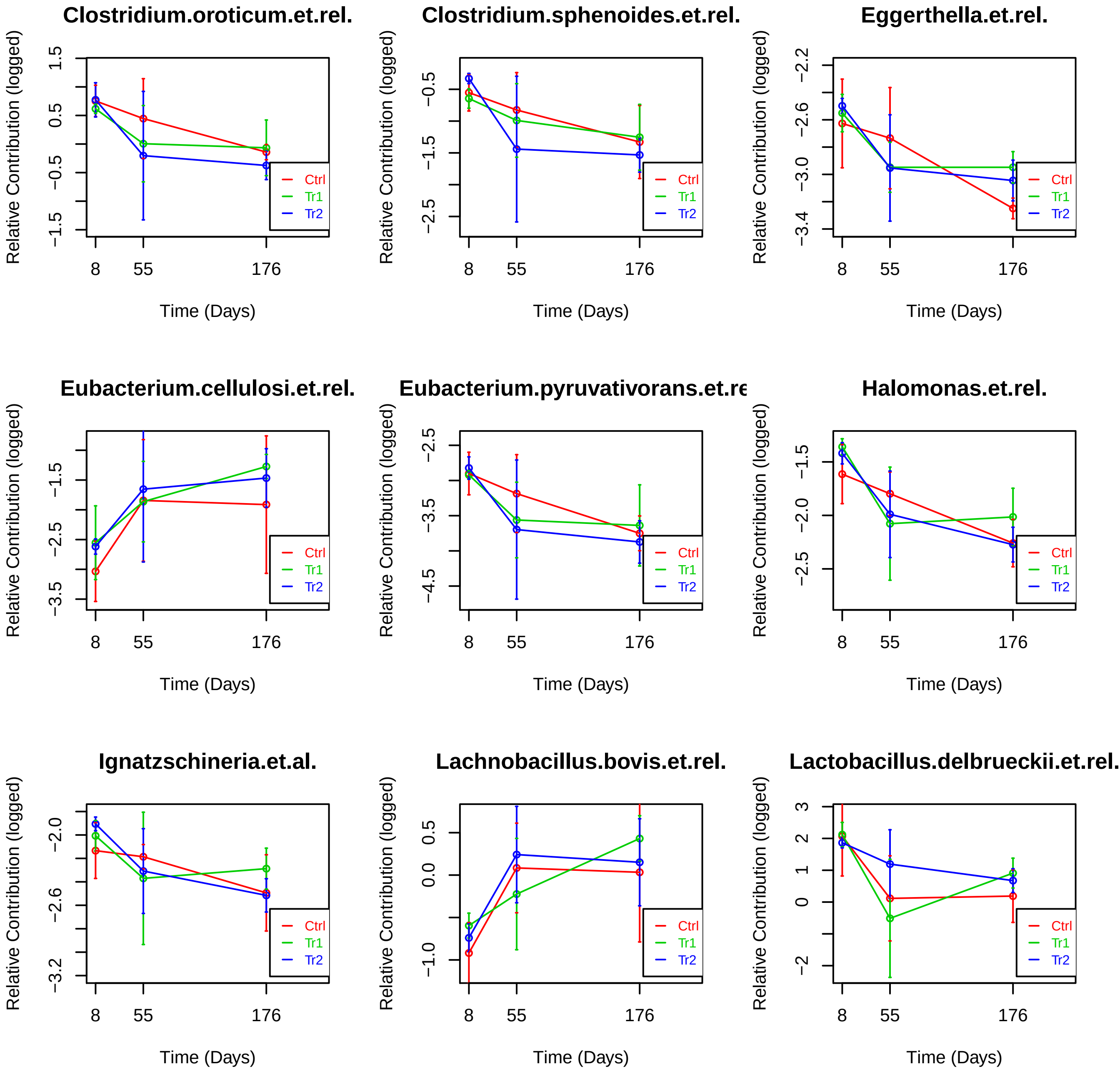

Clostridium.oroticum.et.rel.
Relative Contribution (logged)
1.5
0.5
−0.5
−1.5
8
55
176
Time (Days)
Ctrl
Tr1
Tr2
Clostridium.sphenoides.et.rel.
Relative Contribution (logged)
−0.5
−1.5
−2.5
8
55
176
Time (Days)
Ctrl
Tr1
Tr2
Eggerthella.et.rel.
Relative Contribution (logged)
−2.2
−2.6
−3.0
−3.4
8
55
176
Time (Days)
Ctrl
Tr1
Tr2
Eubacterium.cellulosi.et.rel.
Relative Contribution (logged)
−1.5
−2.5
−3.5
8
55
176
Time (Days)
Ctrl
Tr1
Tr2
Eubacterium.pyruvativorans.et.re
Relative Contribution (logged)
−2.5
−3.5
−4.5
8
55
176
Time (Days)
Ctrl
Tr1
Tr2
Halomonas.et.rel.
Relative Contribution (logged)
−1.5
−2.0
−2.5
8
55
176
Time (Days)
Ctrl
Tr1
Tr2
Ignatzschineria.et.al.
Relative Contribution (logged)
−2.0
−2.6
−3.2
8
55
176
Time (Days)
Ctrl
Tr1
Tr2
Lachnobacillus.bovis.et.rel.
Relative Contribution (logged)
0.5
0.0
−1.0
8
55
176
Time (Days)
Ctrl
Tr1
Tr2
Lactobacillus.delbrueckii.et.rel.
Relative Contribution (logged)
3
2
1
0
−2
8
55
176
Time (Days)
Ctrl
Tr1
Tr2

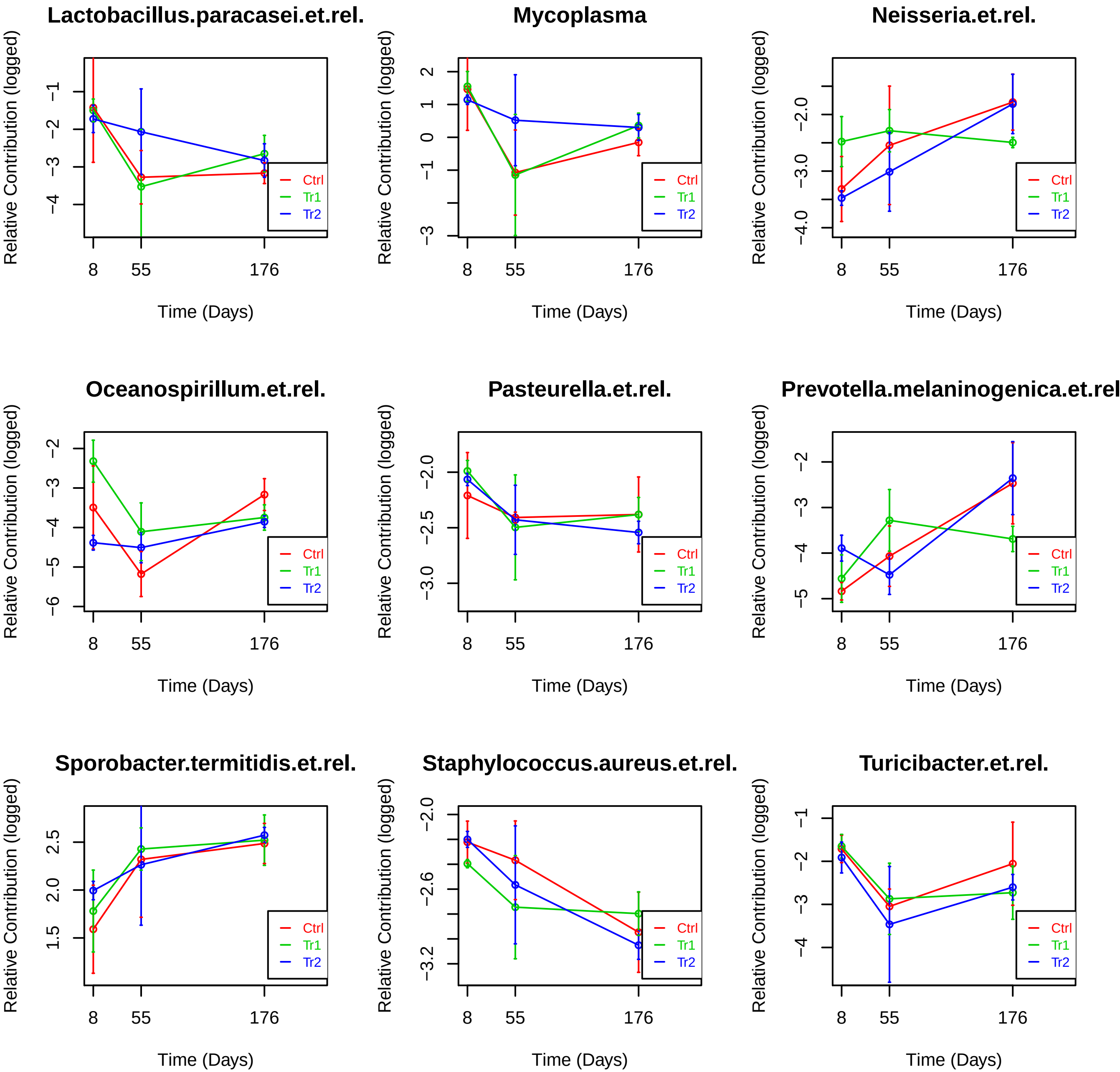
Lactobacillus.paracasei.et.rel.
Mycoplasma
Neisseria.et.rel.
Oceanospirillum.et.rel.
Pasteurella.et.rel.
Prevotella.melaninogenica.et.rel
Sporobacter.termitidis.et.rel.
Staphylococcus.aureus.et.rel.
Turicibacter.et.rel.
Relative Contribution (logged)
Time (Days)
8
55
176
Ctrl
Tr1
Tr2

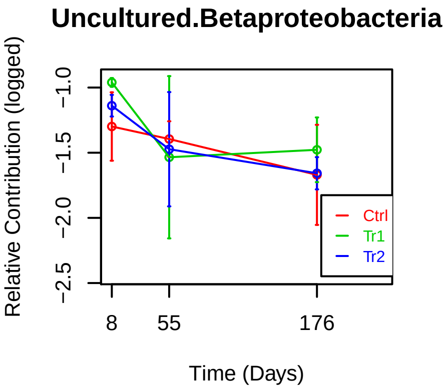

Uncultured.Betaproteobacteria
Relative Contribution (logged)
−1.0
−1.5
−2.0
−2.5
8
55
176
Time (Days)
Ctrl
Tr1
Tr2
